# Controlling Silicification on DNA Origami with Polynucleotide Brushes

**DOI:** 10.1021/jacs.3c09310

**Published:** 2023-12-20

**Authors:** Shuang Wang, Po-An Lin, Marcello DeLuca, Stefan Zauscher, Gaurav Arya, Yonggang Ke

**Affiliations:** † Wallace H. Coulter Department of Biomedical Engineering, Georgia Institute of Technology and Emory University, Atlanta, Georgia 30322, United States; ‡ Department of Mechanical Engineering and Materials Science, Duke University, Durham, North Carolina 27708, United States

## Abstract

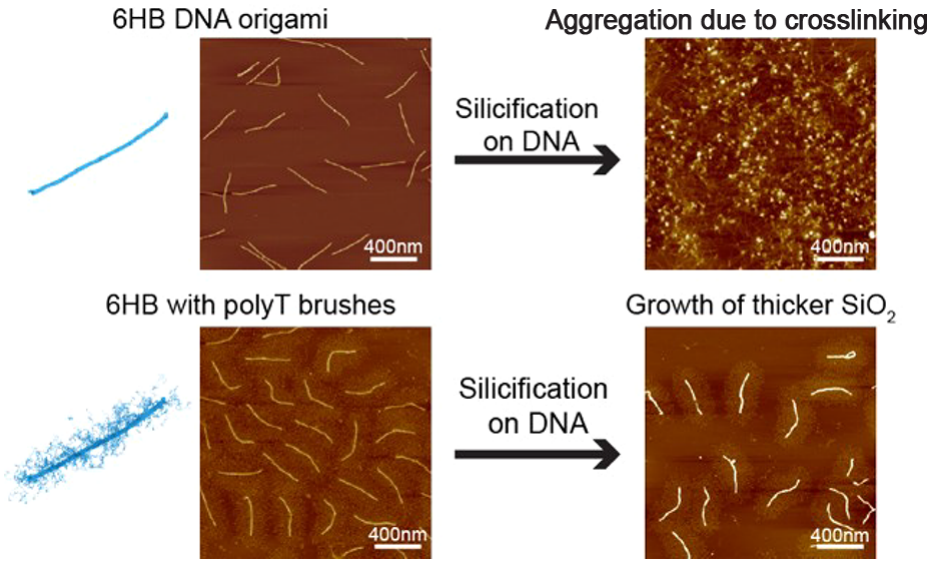

DNA origami has been used as biotemplates for growing a range of inorganic materials to create novel organic–inorganic hybrid nanomaterials. Recently, the solution-based silicification of DNA has been used to grow thin silica shells on DNA origami. However, the silicification reaction is sensitive to the reaction conditions and often results in uncontrolled DNA origami aggregation, especially when growth of thicker silica layers is desired. Here, we investigated how site-specifically placed polynucleotide brushes influence the silicification of DNA origami. Our experiments showed that long DNA brushes, in the form of single- or double-stranded DNA, significantly suppress the aggregation of DNA origami during the silicification process. Furthermore, we found that double-stranded DNA brushes selectively promote silica growth on DNA origami surfaces. These observations were supported and explained by coarse-grained molecular dynamics simulations. This work provides new insights into our understanding of the silicification process on DNA and provides a powerful toolset for the development of novel DNA-based organic–inorganic nanomaterials.

## Introduction

DNA nanotechnology has seen booming development in recent decades, especially since the invention of DNA origami.^1−5^ DNA origami provide a powerful platform for DNA-based nanofabrication,^6^ due to their excellent structural programmability at the nanoscale^7,8^ and the ease with which they can be chemically modified.^9^ For instance, DNA nanostructures have been used as pegboards to assemble other materials, including nanoparticles,^10−12^ carbon nanotubes,^13−15^ and polymer complexes,^16^ as biomolds to grow inorganic nanoparticles with designed sizes and shapes;^17^ and as templates to grow inorganic materials^18−24^ via biomineralization on DNA. The growth of silica on DNA templates (silicification), in particular, has been studied extensively in recent years because the process is relatively easy to control and can significantly improve the mechanical strength and stability of DNA nanostructures.^25^

**Figure 1:**
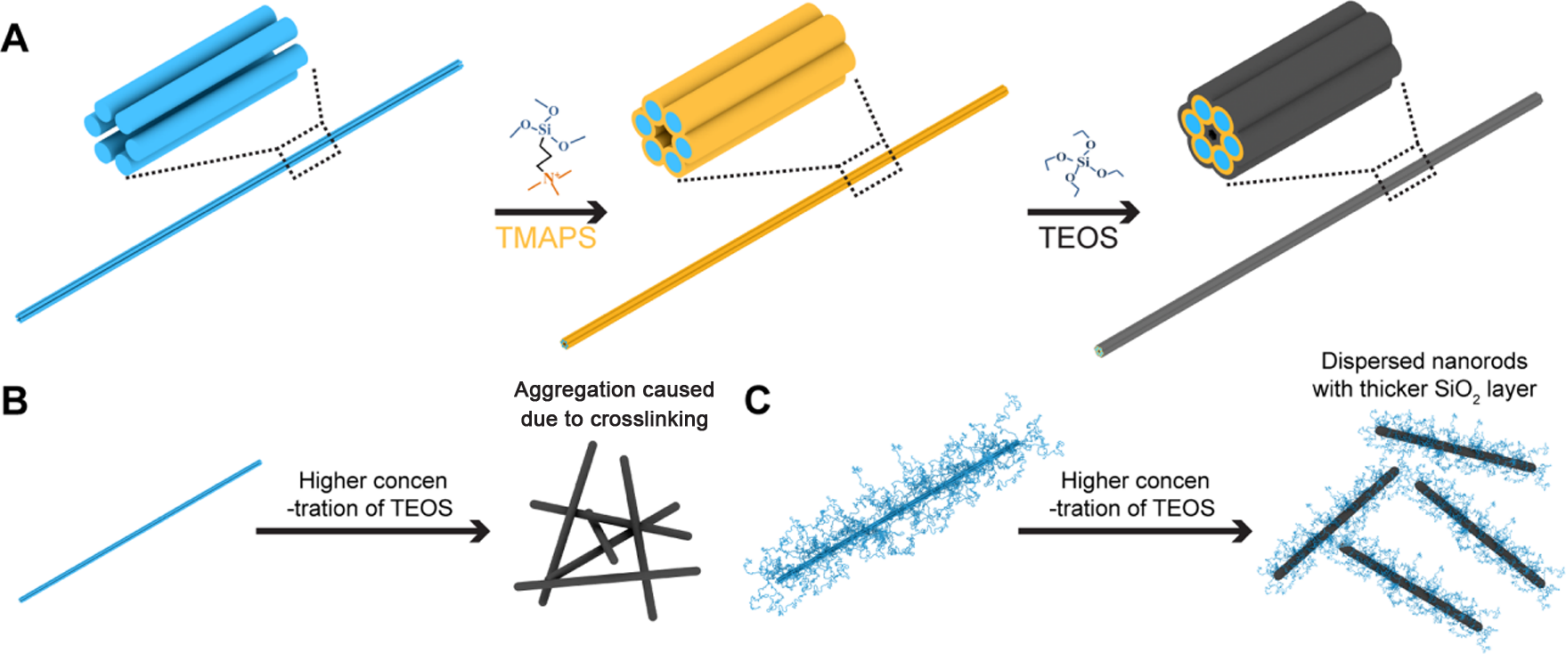
Schematics showing silicification on DNA origami with and without single-stranded DNA brushes. (A) Silicification reaction on a DNA origami template using the Stöber method. (B) High concentrations of reagents generally lead to the aggregation of DNA origami. (C) Long, single-stranded DNA brushes on the origami surface are thought to reduce aggregation and allow for the robust growth of thicker silica shells on DNA origami.

To date, two silicification strategies have been reported. One involves adsorbing DNA nanostructures on a substrate and then reacting them with prehydrolyzed silica clusters produced by mixing precursor *N*-trimethoxysilylpropyl-*N*,*N*,*N*-trimethylammonium chloride (TMAPS) or 3-aminopropyl triethoxysilane (APTES) with tetraethyl orthosilicate (TEOS). However, this “reaction-on-surface method” is slow and often requires the recovery of the silicified DNA nanostructures from the substrate surface to render them useful for applications.^26^ The other method is the sol–gel chemistry-based Stöber method, which has been used to grow silica shells on DNA origami in solution.^22^ This method entails a two-step reaction. In the first step, the positively charged quaternary ammonium group in TMAPS binds to the anionic DNA phosphate backbone. In the second step, hydrolysis and cocondensation of the siloxane group in TMAPS and TEOS forms connective siloxane bridges to produce silica shells (Figure 1A). While the Stöber method works well for growing a thin layer (e.g., ∼2 nm) of silica on DNA origami,^27^ it typically leads to aggregation and hence lower yield, especially for DNA origami purified with polyethylene glycol precipitation,^28^ when higher reactant concentrations are used to generate thicker silica shells. This is due to TEOS-induced cross-linking between adjacent origami (Figure 1B).

Minimizing the TEOS cross-reaction between silica shells during silicification should allow for more robust silica growth and improved tunability of the shell thickness. Recently, it was shown that thicker silica growth could be achieved by coating DNA origami with poly(ethylene glycol)-*b*-poly(l-lysine) block copolymers via electrostatic adsorption.^29^ We therefore hypothesize that a protective “shield” of long DNA strands on the origami surface can effectively prevent the origami cores (where we believe most of the silica growth occurs) from touching and reacting with each other. To achieve this, we turned to a surface-initiated terminal deoxynucleotidyl transferase (TdT) polymerization reaction on DNA origami that we have recently developed.^30^ This reaction can generate single-stranded DNA brushes of relatively uniform, tunable lengths from designable locations on DNA origami surfaces. The flexible single-stranded polynucleotide chains of the brush form a loose and sterically repulsive protective layer on DNA origami. While this layer still allows TMAPS and TEOS to permeate through to reach the DNA origami core for silicification, it also prevents TEOS-induced aggregation of adjacent origami (Figure 1C). This new method enables a significantly more robust silicification of DNA origami and can be used in a range of applications, especially when higher yield and growth of thicker silica shells are required.

## Results

**Figure 2:**
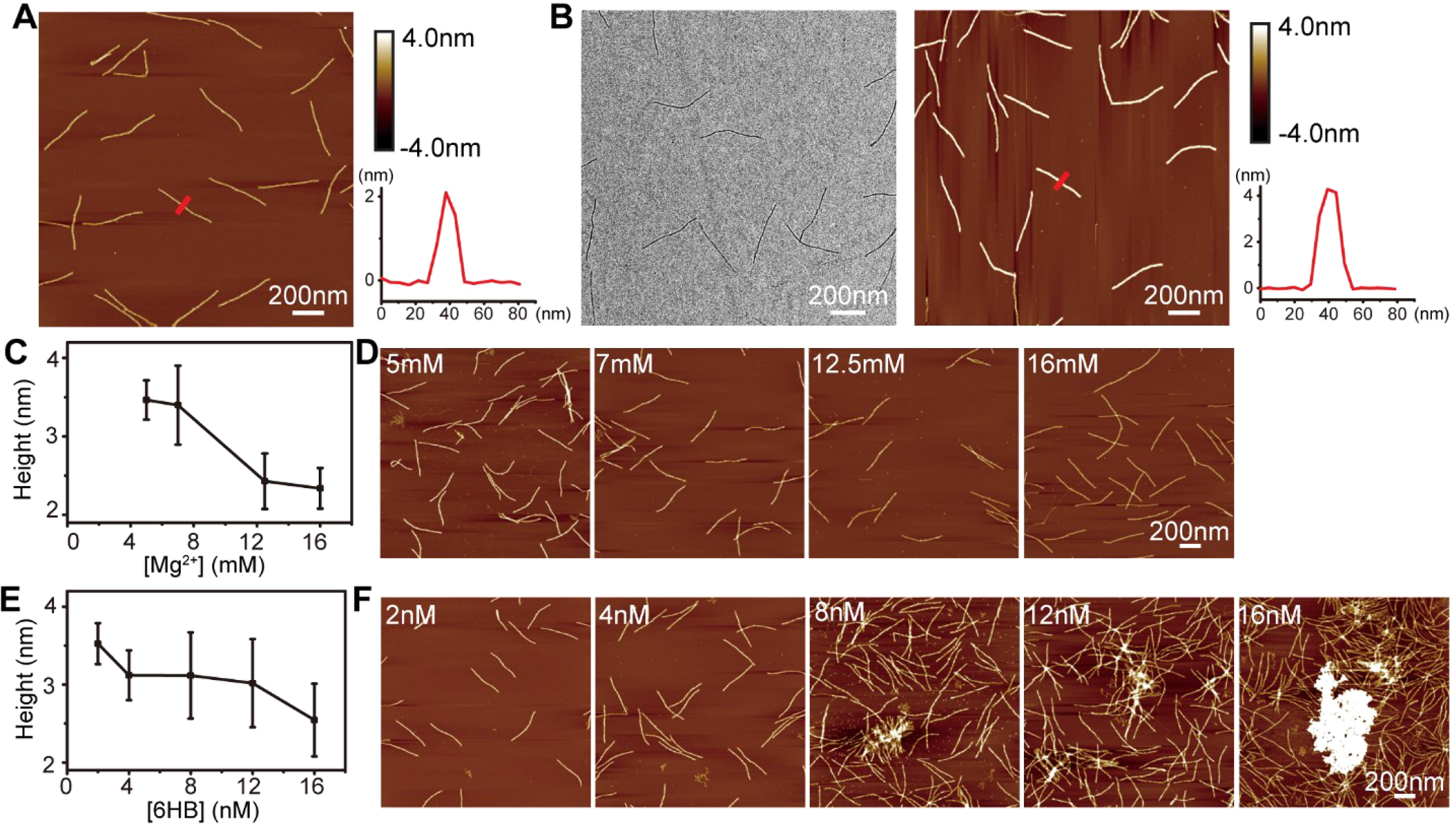
Optimization of the DNA origami silicification reaction conditions. (A) AFM image of the 6HB DNA origami imaged under ambient conditions. The height of the 6HB, determined from AFM images, is ∼2 nm. (B) TEM and AFM images of the 6HB after silicification. After 4 days of reaction, the 6HB height increased to ∼4 nm. [6HB] and [Mg^2+^] concentrations are 2 nM and 5 mM, respectively. (C, D) Effect of [Mg^2+^] concentration (5, 7, 12.5, 16 mM) on the silicification reaction. The measured height of 6HB decreased significantly at higher Mg^2+^ concentrations under a [6HB] concentration of 2 nM after silicification for 3 days. (E, F) Effect of DNA origami concentration on silicification. With increasing concentrations of the 6HB DNA origami, the measured height of the 6HB decreased, and more aggregation occurred. [Mg^2+^] concentration is 5 mM. Reaction time is 3 days. 90 structures were measured for each data point in parts C and E.

For all experiments in this study, we used 400 nm long six-helix bundle (6HB) DNA origami. This DNA nanostructure was designed, synthesized, and subsequently modified with polynucleotide brushes, by using methods published in our previous work.^30^ Successful assembly of the 6HBs was verified with TappingMode atomic force microscopy (AFM, ScanAsyst, Bruker) imaging in air. The AFM images showed the presence of ∼400 nm long, rod-like 6HB nanostructures (Figure 2A). We determined the height of these 6HBs to be ∼2 nm, i.e.., significantly smaller than the theoretical 6 nm theoretical value. We attribute this discrepancy to the significant shrinkage of the 6HB in ambient conditions and to possible compression during the AFM imaging process.^31−33^ After silicification in solution, AFM imaging of the 6HB under ambient conditions showed that the 6HB height increased to ∼4 nm, suggesting that a ∼1 nm thick silica shell had grown on the surface of the DNA origami (Figure 2B). The presence of the silica shell was also verified by transmission electron microscopy (TEM) without staining (Figure 2B).

Next, we investigated how the ionic strength affects the silicification on 6HBs. Mg^2+^ ions are commonly included during the self-assembly of DNA nanostructures in solution because divalent cations mitigate the electrostatic repulsion between negatively charged DNA double helices. However, Mg^2+^ can also compete with TMAPS for binding to the negatively charged DNA backbone and therefore reduce the efficiency of silicification.^22^ We examined the effect of the Mg^2+^ concentration [Mg^2+^] ranging from 5 to 16 mM on the silicification (Figure 2C). The 6HB origami samples were initially assembled in a buffer containing 12.5 mM Mg^2+^, and the [Mg^2+^] was then adjusted to the desired concentration via buffer exchange. We found that a low [Mg^2+^] is conducive for silica growth, as shown in Figure 2C,D, where the height of the 6HB was ∼3.5 nm (*n* = 90) at both 5 and 7 mM Mg^2+^. The findings were consistent with previously reported results.^22^ However, when we increased the [Mg^2+^] from 7 to 12.5 mM, the silica growth decreased significantly (Figure 2C,D). Low [Mg^2+^] concentrations are generally insufficient to maintain the integrity of DNA origami nanostructures. However, silica growth at 5 mM Mg^2+^, the lowest [Mg^2+^] we tested, stabilized the origami and, compared to higher [Mg^2+^], yielded the most homogeneous silica shell growth (Figure 2C, height measurement in Supporting Information Figure S1). Therefore, we chose to work with 5 mM Mg^2+^ for all subsequent experiments.

We also tested the effect of changes in the 6HB origami concentration on the silicification reaction. When keeping concentrations of TMAPS and TEOS constant, we observed a decrease in the thickness of the silica shell with increasing concentration of 6HB (Figure 2E). We attribute this to the reduced precursors per [6HB]. At higher 6HB concentrations we also observed a higher degree of aggregation (Figure 2F), likely due to more extensive cross-linking between the silica shells on adjacent 6HBs, which was also observed in previous work.^22^

Finally, we tested the effects of different TMAPS and TEOS concentrations on the silicification process. In these experiments, we used a relatively low 6HB origami concentration of 2 nM, compared with previous works.^22^ This allows us to use higher precursor/DNA ratios without triggering aggregation. First, we varied the TMAPS concentration while keeping the TEOS concentration constant. We found that the thickness of the silica shell increased with increasing [TMAPS], until it reached its peak value at a [6HB]/[TMAPS] ratio of 1/225 (height vs [TMPAS], Supporting Information Figure S2A). [6HB]/[TMAPS] means the molar ratio between phosphate groups in 6HB and TMAPS molecules. The shell thickness started to decrease when [TMAPS] was further increased to yield [6HB]/[TMAPS] ratios of 1/270 and 1/300. We believe that this behavior is due to the saturation of the origami surface with TMAPS at the optimal 1/225 ratio and that an increase in the [TMAPS] beyond this threshold value results in an increasing amount of unbound TMAPS in solution (schematics in Supporting Information Figure S2B). This in turn leads to an increase of nontemplated silicification in solution, which also consumes TEOS. Next, we tested the effect of increasing the TEOS concentration while maintaining a constant [6HB]/[TMAPS] ratio of 1/225 (height vs [TEOS], Supporting Information Figure S2C). In this case we observed a substantial increase in the silica shell thickness when the ratio of [6HB]/[TEOS] was halved, from 1/60 to 1/120. However, a further decrease of the [6HB]/[TEOS] to 1/135 resulted in a decrease in shell thickness. A possible mechanism for this reduction in shell thickness is that very high concentrations of TEOS also induce more nontemplated silicification reactions in solution that compete for reactants with the 6HB-templated silicification (schematics in Supporting Information Figure S2D).

So far, our silicification experiments on the 6HB produced only thin silica shells of up to ∼1 nm in thickness, even when using optimal Mg^2+^, TMAPS, and TEOS concentrations. Increasing the concentrations of salt or reactants, or prolonging the reaction time, only led to increased aggregation without generating thicker silica shells on the DNA origami. To overcome this limitation, we decided to create site-specific, sterically repulsive surface modifications on the DNA origami by growing long (hundreds of bases), single-stranded polythymine (polyT) chains on the origami surfaces. To achieve this, we harnessed TdT-catalyzed enzymatic polymerization (TcEP), a surface-initiated, enzymatic polymerization reaction we developed previously.^30,34,35^ Unlike double-stranded DNA, which has a well-defined, rod-like conformation, single-stranded DNA is conformationally much more flexible. Additionally, there is a significant difference in the surface charge distribution between double-stranded DNA and single-stranded DNA, and some studies have shown that silicification occurs preferentially on DNA duplexes rather than on single-stranded DNA.^36,37^ We thus hypothesized that, in addition to providing steric repulsion, the polyT brushes on 6HB are much less likely to interact with TMAPS and thus effectively reduce 6HB cross-linking during silicification.

**Figure 3:**
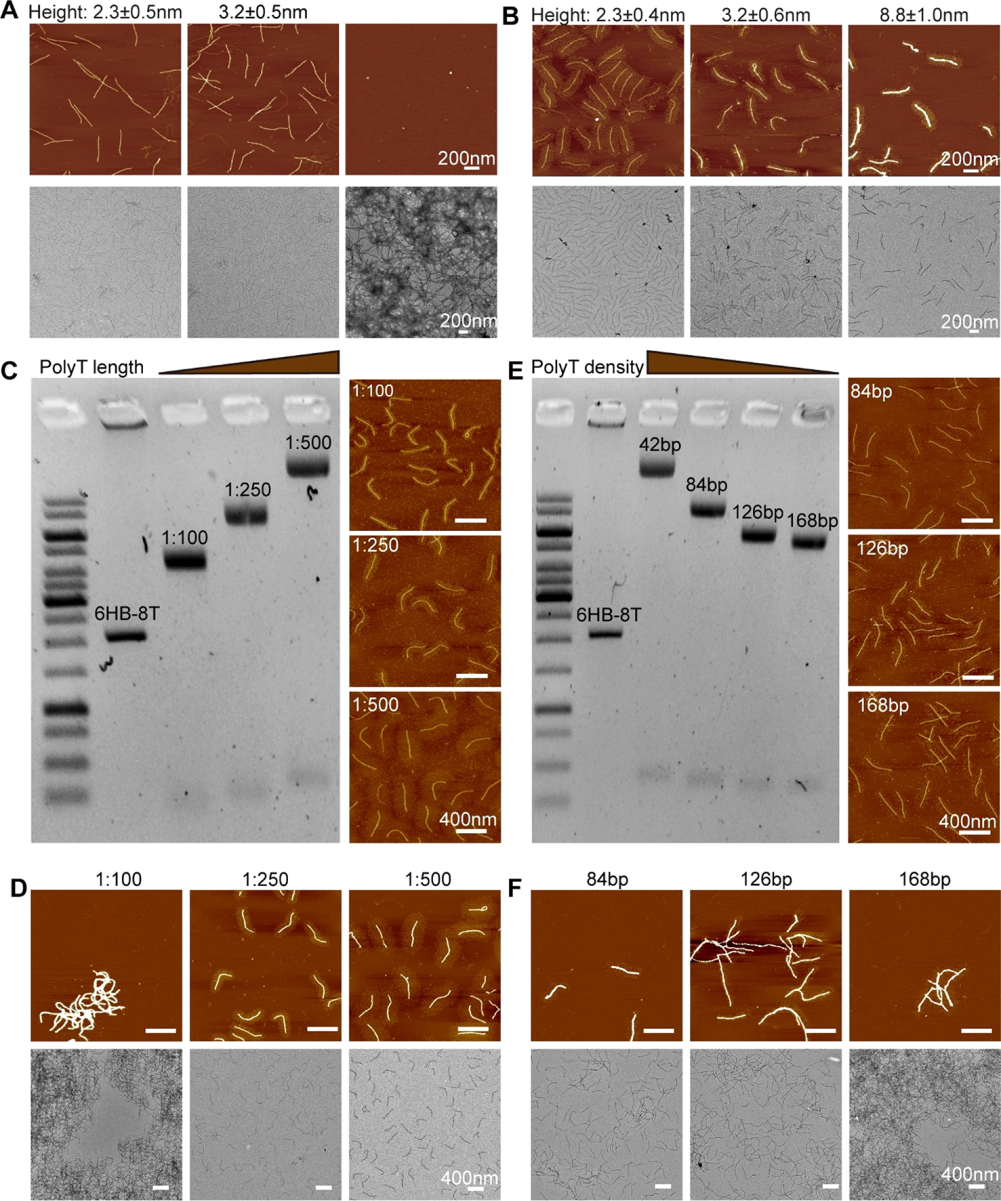
Effect of polyT brushes on the silicification process. (A) AFM and TEM images of unmodified 6HB and of (B) 6HB-27*-SS after silicification over 1, 2, and 4 days. (C) Agarose gel and AFM images of 6HB-27*-SS with controllable length grown by varying the [initiator]/[dTTP] feed ratios (1:100, 1:250, 1:500). (D) AFM and TEM images of 6HB-27*-SS with different lengths of polyT after silicification for 4 days. *C*_6HB_ = 2 nM, *C*_TMAPS_ = 2 mM, and *C*_TEOS_ = 1.4 mM. (E) Agarose gel and AFM images of 6HB-27*-SS with programmable graft density (i.e., 84 bp, 126 bp, and 168 bp distance between two closest neighboring initiators). (F) AFM and TEM images of 6HB-27*-SS with different polyT graft densities after silicification for 4 days. *C*_6HB_ = 2 nM, *C*_TMAPS_ = 2 mM, *C*_TEOS_ = 1.4 mM.

To test this hypothesis, we compared the silicification achieved on 6HB (Figure 3A) with that achieved on 6HBs modified with 162 polyT strands, evenly distributed over the full length of the 6HB (Figure 3B). The design of the 6HB was divided into 27 domains along its length, each containing six possible positions where polyT can grow. This is shown schematically in Figure S3, where an asterisk (*) indicates a domain that is modified with six polyT strands. For example, the 6HB-27*-SS is a 6HB structure whose 27 domains are completely covered with polyT single strands, and a 6HB-5*/17/5*-SS is a 6HB structure with 17 unmodified domains in the middle and 5 domains, each modified with polyT strands, at both ends of the 6HB. The heights of the 6HB and the 6HB-27*-SS obtained by AFM imaging in air were similar before silicification, likely because the flexible polyT strands were completely flattened on the origami and mica substrate surface. After exposing the origami to the silicification conditions for 1 day, the dry heights of both 6HB and 6HB-27*-SS increased by ∼0.3 nm (Figure 3B, TEM images in Supporting Information Figure S4. This observation suggests that the polynucleotide brushes on the origami surface had only a negligible influence on the silicification of the 6HB core. After the origami was exposed to the silicification conditions for 2 days, the heights of both 6HB and 6HB-27*-SS increased by ∼0.9 nm, still with only a negligible height difference between the two samples. However, close inspection of TEM images indicated that a small amount of aggregation had occurred for the 6HB samples, while no aggregation was visible for the 6HB-27*-SS sample. Compared to room temperature, silicification on 6HB at a higher temperature of 35 °C led to more aggregation after two-day incubation (AFM images in Supporting Information Figure S5). This result is consistent with what was reported in previous work,^36^ suggesting higher temperature significantly increases kinetics of silicification on DNA origami and thus elevates aggregation. When silicification was extended to 4 days, we observed a clear height difference between the two samples. The average height of the 6HB-27*-SS origami increased to ∼8.8 nm, suggesting that a thick silica shell (∼3.4 nm) had grown on its surface. Furthermore, although all 6HB samples showed some aggregation after 4 days of silicification, many individual 6HB-27*-SS nanorods could still be observed in the AFM and TEM images. In contrast, no observable changes occurred to the conformation of the polyT brushes during the silicification reaction, indicating that there was only minimal if any silica growth on single-stranded polynucleotide strands. Together, these results suggest that polyT brushes can indeed reduce origami aggregation during the silicification process and facilitate the growth of substantially thicker silica shells on DNA nanostructures.

Next, we investigated the influence of the polyT brush length and grafting density on the silicification process. To control the length of the polyT brushes, we maintained the number of initiators on 6HB constant and varied the [initiator]/[dTTP] ratios from 1/100 to 1/500. Agarose gel electrophoresis showed that the mobility of the reaction products decreased with increasing dTTP concentration, indicating brush growth, which was also directly confirmed by the AFM images (Figure 3C). When subjected to the silicification process, the 6HB with short brushes (1/100) aggregated significantly, while 6HB with longer brushes (1/250,1/500) remained dispersed in the solution (Figure 3D). In the original design of 6HB-27*-SS, the distance between two closest neighboring initiators on the same DNA duplex is 42bp, which was sufficiently effective in preventing aggregation. To study the effect of brush grafting density, we designed three new structures with reduced initiator densities (i.e., 84, 126, and 168 bp distances between two closest neighboring initiators; see Supporting Information Figure S6 for more details). Gel electrophoresis showed that the reaction products moved faster with decreasing polyT brush graft density; the density of polyT brushes was also visible in the AFM images (Figure 3E). As expected, under the same silicification conditions, the degree of cross-linking was exacerbated with decreasing graft density (Figure 3F).

**Figure 4:**
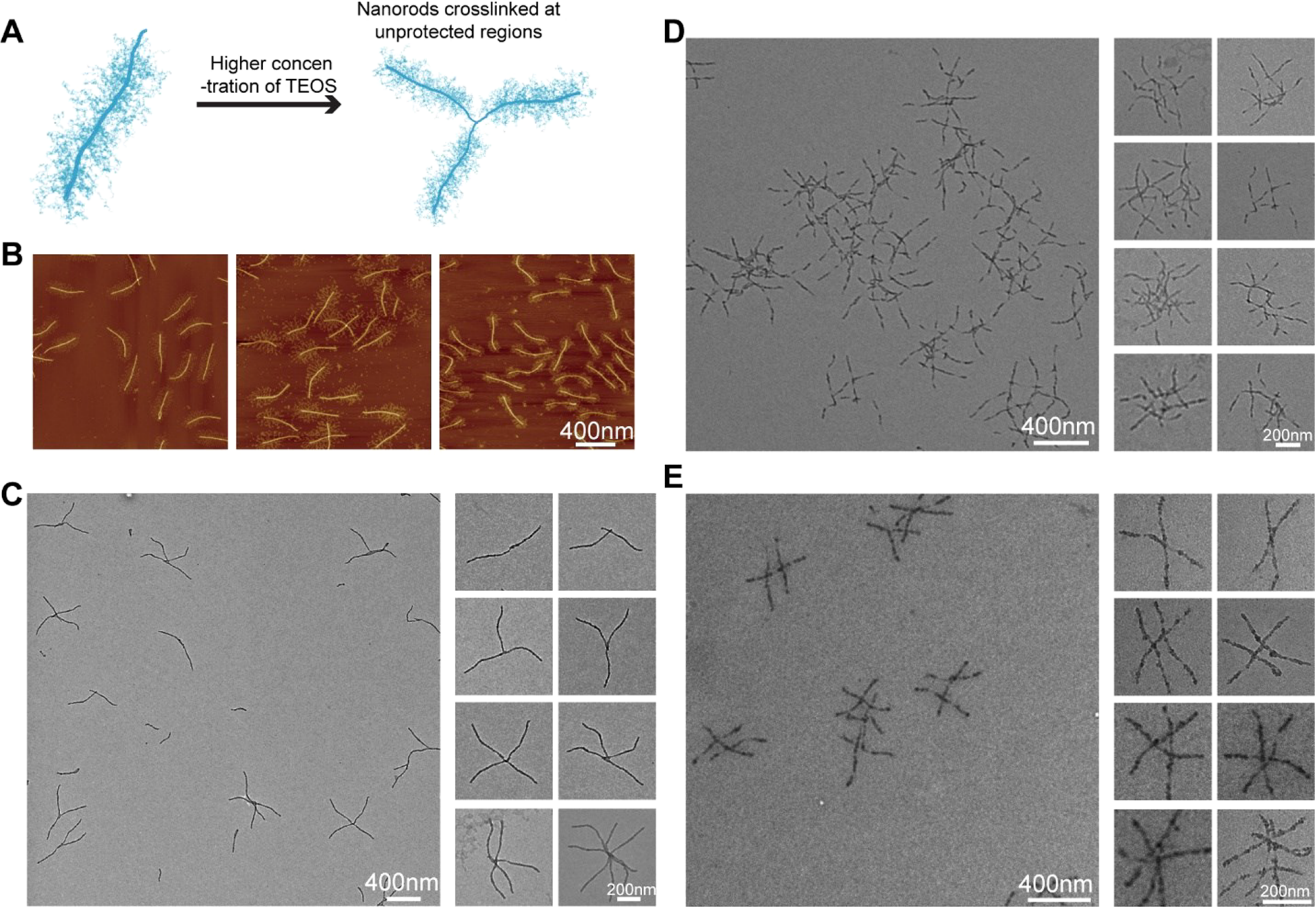
Silicification of 6HB modified with polyT brushes. (A) Silicification on 6HB partially protected with brushes leads to origami cross-linking through precursor-induced silica bridges at exposed, unprotected sections on the origami. (B) AFM images of a 6HB-18*/9-SS, a 6HB-9*/18-SS, and a 6HB-5*/17/5*-SS. (C–E) TEM images of 6HB-18*/9-SS and 6HB-9*/18-SS and of 6HB-5*/17/5*-SS after silicification, respectively.

Encouraged by these results, we hypothesized that it should be possible to modulate DNA origami cross-linking by only partially modifying the origami with polyT brush, which should enable the creation of novel DNA origami superstructures (Figure 4A). To test this hypothesis, we synthesized 6HB structures that were only partially protected by polyT brushes. Specifically, we synthesized three variants: a 6HB-18*/9-SS with a relatively small, unprotected end section, a 6HB-9*/18-SS with a large, unprotected end section, and a 6HB-5*/17/5*-SS with an unprotected area in the middle of the rod (Figure 4B, AFM images in Supporting Information Figure S7). Silicification reactions were carried out on all three samples until obvious cross-linking was observed. The results shown in Figure 4C–E and TEM images in Supporting Information Figures S8–S13 largely confirmed our hypothesis. For the 6HB-18*/9-SS structure, cross-linking occurred only at or close to the unprotected ends, which resulted in star-like dimers, trimers, tetramers, and a small number of larger oligomers (Figure 4C, Supporting Information Figures S8 and S9). Because of the larger unprotected section at one end, the 6HB-9*/18-SS produced primarily oligomeric clusters that typically contained more than four monomeric nanostructures (Figure 4D, Supporting Information Figures S10 and S11). Silicification of the 6HB-5*/17/5*-SS also generated oligomeric clusters, but the cross-linking clearly took place at the unprotected middle section of the nanorods (Figure 4E, Supporting Information Figures S12 and S13).

Next, to better understand the cross-linking process, we studied the silicification on 6HB-5*/17/5*-SS with different concentrations of TEOS (TEM images in Supporting Information Figure S14). At low [TEOS] (0.3 mM), a thin silica shell was grown on 6HB-5*/17/5*-SS and no cross-linking was observed. Cross-linking started to appear at a [TEOS] of 1 mM, even though the 6HB-5*/17/5*-SS were still mostly present as monomers. When we doubled the TEOS concentration to 2 mM, all monomers disappeared and only oligomeric clusters were seen in TEM images. These results prove that the formation of oligomeric, self-assembled origami clusters arises due to cross-linking during the silicification process.

**Figure 5:**
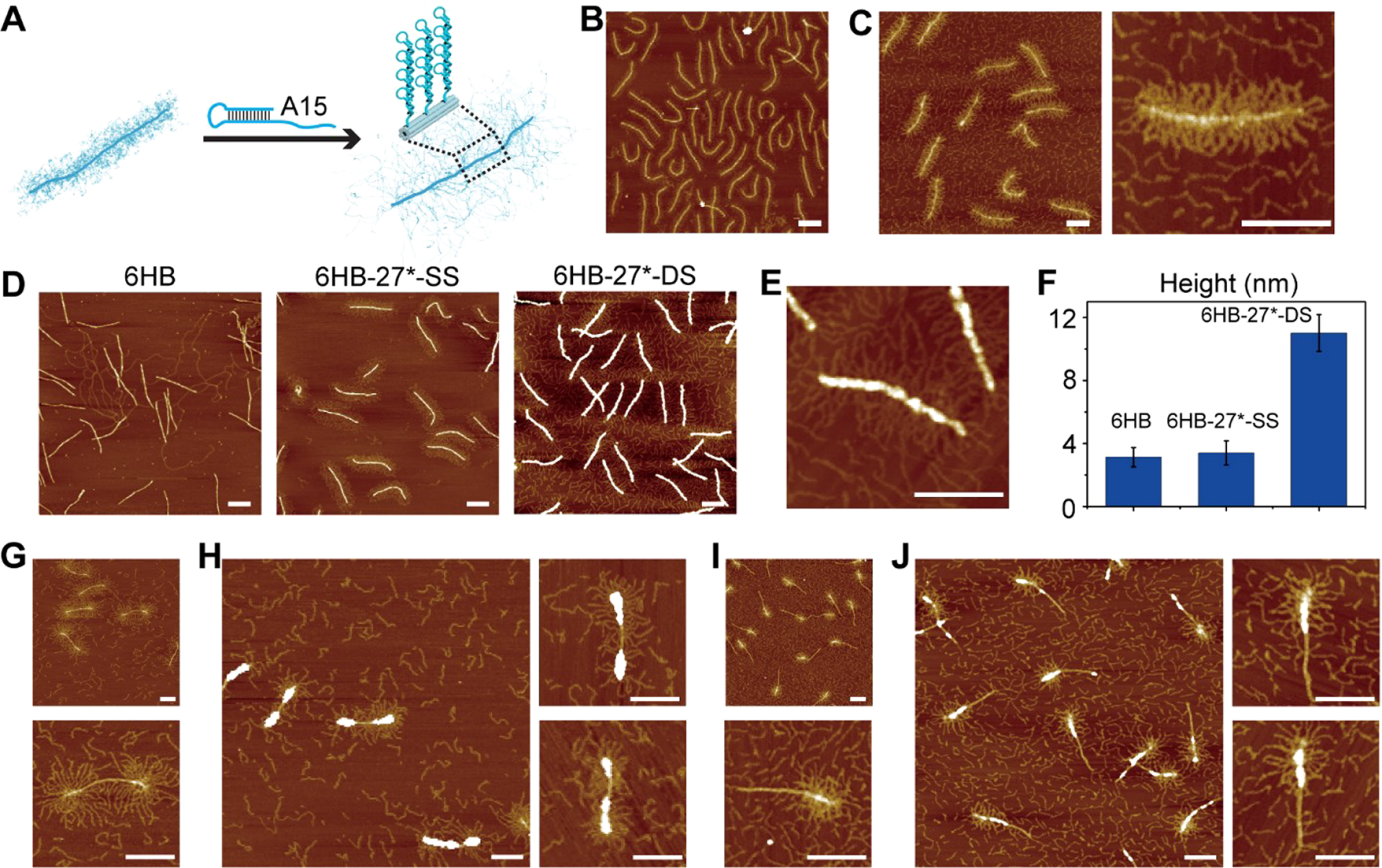
Study of silicification of 6HB modified with double-stranded DNA brushes. (A) Single-stranded polyT brushes are largerly converted to double-stranded brushes by adding a hairpin DNA strand with a complementary A15 hybridization domain. (B, C) AFM images of 6HB-27*-SS before and after the addition of the hairpin DNA strand. (D) AFM images of 6HB, 6HB-27*-SS, and 6HB-27*-DS after silicification. (E) Zoomed-in AFM image of 6HB-27*-DS after silicification showing significant silica growth on the 6HB core. (F) Comparison of heights of 6HB, 6HB-27*-SS, and 6HB-27*-DS after silicification. (G, H) AFM images of 6HB-5*/17/5*-DS before and after silicification. (I, J) AFM images of 6HB-9*/18-DS before and after silicification. All scale bars are 200 nm.

It was recently reported that short DNA duplexes on DNA origami surfaces promote silicification.^36^ Therefore, we sought to investigate the effect of long, double-stranded (DS) DNA brushes on the silicification process as compared to the single-stranded polyT brushes we used above. To convert single-stranded polyT brushes to double-stranded brushes, we added an excess of DNA strands that contained a hairpin with a complementary, 15-adenine single-stranded domain, to the reaction mixture (Figure 5A). Although it is unlikely that all polyT brushes would be converted to double strands, we expected that a sufficiently large number of polyT single-stranded brushes would be converted to induce a change in brush morphology due to the significantly higher rigidity of double-stranded DNA. In AFM images, the double-stranded brushes indeed appear to be extended and more visible than the single-stranded polyT brushes (Figure 5B,C, AFM images in Supporting Information Figure S15). Interestingly, three-day silicification on 6HB-27*-DS led to significantly enhanced silica growth on the 6HB core as compared to the growth on 6HB or 6HB-27*-SS, while aggregation caused by cross-linking was still suppressed (Figure 5D,E, AFM images and height measurements in Supporting Information Figure S16). AFM height measurements in Figure 5F showed that the heights of 6HB-27*-DS increased to 11.0 ± 1.16 nm (*n* = 30), while those of 6HB and 6HB-27*-SS increased to only 3.13 ± 0.62 and 3.39 ± 0.77 nm (*n* = 30), respectively. To further establish that the presence of double-stranded DNA brushes induced this substantial increase of silicification, we also performed silicification reactions on 6HB-5*/17/5*-DS and 6HB-9*/18-DS DNA nanostructures (Figure 5G–J, AFM images in Supporting Information Figures S17 and S18). In both nanostructures, silicification was clearly stimulated at the positions with DS brushes compared to the exposed sections of 6HB. While there may have been a small degree of silica growth also on the double-stranded brushes, it was not detectable in the AFM images. In previous work,^36^ it was observed that silica preferetially grew at locations where short DNA duplexes were attached. Our AFM images showed the silicification primarily takes place on or very close to the 6HB DNA origmai surface, when using long, double-stranded DNA brushes. In both cases, it is likely that the preferetial growth is driven by the increased density of negative charges. In summary, our data suggest that double-stranded DNA brushes selectively promote silicification on brush-covered DNA origami surfaces, while not significantly influencing the silification reaction kinetics. Furthermore, the surface roughness increased with increasing silica shell thickness, which is consistent with the reported nucleation and growth mechanism of monodisperse silica nanoparticles.^38^

**Figure 6:**
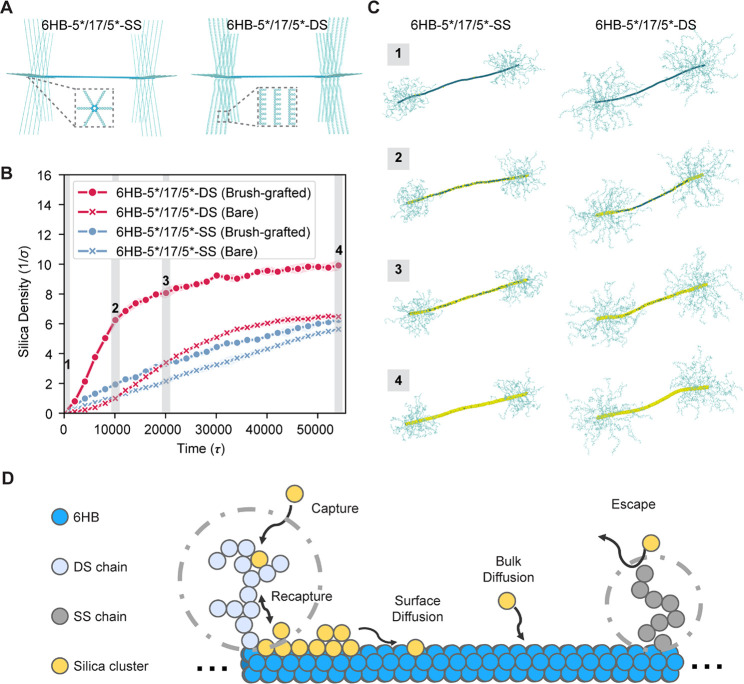
Coarse-grained modeling of silica condensation on brush-functionalized 6HB. (A) Coarse-grained models of the 6HB-5*/17/5*-SS (left) and 6HB-5*/17/5*-DS (right) structures showing close-up views of the bead-chain models adopted for describing single- and double-stranded DNA brushes. (B) Number density of silica precursors condensing on the brush-grafted and bare regions of 6HB-5*/17/5*-SS and 6HB-5*/17/5*-DS. (C) Simulation snapshots corresponding to the labeled time points in (B) with the size of silica precursors doubled for enhanced visibility. (D) Proposed mechanism for enhanced silicification in the brush-functionalized region of a 6HB by double-stranded brushes. Silica precursors are more readily captured and retained by the highly extended and charged double-stranded brushes compared to the conformationally more flexible and weakly charged single-stranded brushes. The adsorbed precursor gradually covers the surface of 6HB through surface diffusion along the chains and the 6HB.

We hypothesized that the surprising effect of double-stranded DNA brushes on DNA origami silicification arises from how these brushes influence the interaction and retention of TMAPS and TEOS on the DNA origami surface. To investigate how DNA brushes influence the condensation of silica on the surface of 6HBs, we carried out molecular dynamics (MD) simulations. To access the long time scales associated with the silicification process, we first developed coarse-grained (CG) models of the 6HB-5*/17/5*-SS structure with single-stranded DNA brushes, the 6HB-5*/17/5*-DS structure with double-stranded DNA hairpin brushes, and the TMAPS-TEOS silica precursors (Figure 6A, coarse-grained model in Supporting Information Figure S19). We then carried out MD simulations of the two brush-functionalized 6HB structures, starting from a similar concentration and distribution of silica precursors in solution, and studied how the precursors condensed and accumulated on the 6HBs as a function of time.

Consistent with our experimental findings, the simulations show that double-stranded brushes promote stronger silicification on the 6HB compared to single-stranded brushes (Figure 6B,C). Specifically, we find that ∼54% more silica precursors condensed on the DS-DNA brush-functionalized regions of 6HB-5*/17/5*-DS compared to its bare region, whereas the corresponding enhancement in condensation by the SS-DNA brushes is only ∼11% in the case of 6HB-5*/17/5*-SS (Figure 6B). Further analysis suggests that the double-stranded brushes likely enhance silicification via two effects: First, the brushes enhance the electrostatic attraction of silica precursors to the 6HB surface due to the intrinsically higher charge density of double-stranded brushes as compared to single-stranded brushes. Our electrostatic energy calculations indicate that 6HB-bound precursors experience ∼2–3 *k*_B_*T* (thermal energy, of magnitude ∼0.6 kcal/mol) stronger attraction with the 6HB surface in the double-stranded brush region compared to the bare or single-stranded brush regions (models and comparison in Figure S20). Second, the brushes facilitate the recruitment and retention of silica precursors at the 6HB surface (Figure 6D). Simulation trajectories in Figure S21 and radial brush charge density calculations in Figure S22 suggest that the precursors are more likely to be captured from solution by the significantly more extended and strongly charged double-stranded DNA brushes. These DS-DNA brushes also help in recapturing silica precursors attempting to leave the 6HB surface. We find that the precursors are retained almost four times longer in the double-stranded brushes compared with their single-stranded counterparts (Figure S21). Also consistent with the experimental results (Figure 2D), our simulations indicate lower silicification rates at higher [Mg^2+^] (simulations in Figure S23).

Next, we investigated whether polyT brushes could improve the silicification of flat DNA origami structures, which are particularly prone to aggregation. A single-layer triangle-shaped DNA origami was synthesized and tested. Before silicification, successful formation of bare triangle origami and polyT-protected triangle origami were confirmed with AFM (Supporting Information Figure S24A,C). After silicification, the bare triangle origami aggregated readily (Supporting Information Figure S24B), while triangle origami with polyT brushes remained well-dispersed. Thickness measurements of brush-modified triangle origami confirmed silica growth (Supporting Information Figure S24D).

While DNA nanostructures are susceptible to high temperature,^39^ studies have shown that coating with inorganic materials can effectively improve thermal stability of DNA origami.^23^ To investigate whether silicification on polyT-brush-coated 6HB DNA origami can improve its thermal stability, 6HB-27*-SS with and without silica shell were incubated at 70 °C for 30 min, and imaged with TEM (images in Supporting Information Figure S25). 6HB-27*-SS without silicification was heavily damaged. In contrast, although defects were observed on some structures. 6HB-27*-SS with silicification was largely intact, maintaining its nanorod geometry. We expected that the growth of the silica shell would increase the stiffness of our 6HB nanorods. To verify this, we used the program Easyworm^40^ (Supporting Information Figure S26) to evaluate the persistence lengths of 6HB DNA origami with and without silica shell. Our analysis showed that the persistence lengths of 6HB-27*-SS increased from 555 ± 246 nm (no silica, close to previously reported values^41^) to 834 ± 110 nm and 1054 ± 165 nm with increasing silica thickness from 0.9 to 3.4 nm, respectively. The increased persistence lengths of silica-coated 6HB-27*-SS indicate that silicification significantly increases the stiffness of DNA origami significantly.

## Conclusions

In this work, we systematically investigated how single- and double-stranded DNA brushes on the surface of DNA origami nanostructures influence silica growth. Our results showed that single-stranded brushes grown on the entire surface of DNA origami efficiently prevent their cross-linking and aggregation during the silicification process, thus providing a useful approach to achieve a more robust silicification on DNA origami. This is particularly useful for growing thicker silica shells (using a longer reaction time without causing aggregation) on DNA origami for improved stability and mechanical properties. We further demonstrated that DNA origamis that are only partially protected by single-stranded DNA brushes readily self-assembled into oligomeric aggregates due to silica-induced cross-linking at unprotected surface domains. This is an exciting observation as site-specific brush modification of DNA origami thus opens a viable avenue for producing controlled micellar and network-like assemblies of silicified DNA origami nanostructures. Finally, we showed that like single-stranded brushes, double-stranded DNA brushes can also suppress cross-linking of DNA origami. In addition, however, we found that the presence of double-stranded brushes significantly promoted silicification on the DNA origami surface. This process is selective, as we observed enhanced silica growth only on the surface domains of DNA origami that were modified with these brushes. Furthermore, we observed that silicification occurs primarily on the surface of DNA origami but not on the long, double-stranded DNA brushes. These phenomena were further investigated by MD simulations, which not only corroborated significantly enhanced accumulation of TMAPS on DNA origami surfaces covered with double-stranded brushes but also showed that this effect arises from a combination of enhanced electrostatic potential close to the brush grafting points and enhanced recruitment and retention of silica precursors at the origami surface. We believe that our controlled, selective silicification method can be readily used to explore and produce DNA-templated organic–inorganic composite materials for various applications.

## Supporting Information Available

Materials, design, annealing and purification protocol of DNA origami, reaction conditions for TdT polymerization and silicification on DNA origami, imaging protocols, simulation methods, and TEM images and AFM images of DNA origami (PDF)
