# Supplementary material for: Controlling Silicification on DNA Origami with Polynucleotide Brushes
Source: J Am Chem Soc. 2023 Dec 20;146(1):358–67. doi: 10.1021/jacs.3c09310 (PMC10785815; doi:10.1021/jacs.3c09310)
Supplement: Supplementary file 1 — ja3c09310_si_001.pdf [file ja3c09310_si_001.pdf]

Supplementary Information

Controlling Silicification on DNA Origami with Polynucleotide Brushes

Shuang Wang[1], Po-An Lin[2], Marcello DeLuca[2], Stefan Zauscher[2], Gaurav Arya[2], Yonggang Ke[1*]

[1]Wallace H. Coulter Department of Biomedical Engineering, Georgia Institute of Technology and Emory University, Atlanta, Georgia 30322, United States
[2]Department of Mechanical Engineering and Materials Science, Duke University, Durham, North Carolina 27708, United States

*yonggang.ke@emory.edu

Materials

The single-stranded DNA scaffold p7308 (used in 6HB) was extracted from M13 bacteriophage. All the short DNA staple strands were synthesized and purchased from Integrated DNA Technologies (www.idtdna.com) and used without any purification. dTTP was purchased from Thermo Fisher Scientific. Terminal deoxynucleotidyl transferase (TdT) including 10× TdT reaction buffer were ordered from Promega. Magnesium chloride hexahydrate ($MgCl_2·6H_2O$), tris (hydroxymethyl)aminomethane (Tris), ethylenediaminetetraacetic acid (EDTA), Ethidium Bromide (EB) were ordered from Sigma-Aldrich. Freeze 'N Squeeze spin column and Amicon centrifugal filter (100 kDa, 10 kDa, MWCO, 0.5 mL) were purchased from Bio-Rad (USA) and Merck Millipore, respectively. Agarose was ordered from Benchmark Scientific. AFM SCANASYST Mode™ silicon cantilevers (kf = 40 N/m, freq = 311-357 kHz, RTip < 10 nm) were ordered from Bruker. Uranyl formate powder and CF400-CU EM grids were purchased from Electron Microscopy Sciences. 0.2 μm syringe filters were bought from VWR International.

Design, fabrication and purification of DNA origami

Six helix bundle (6HB) were designed by caDNAno software (http://cadnano.org/) and synthesized by mixing P7308 scaffold strand (10 nM) and unpurified DNA strands (100 nM) in 1×TE buffer (40 mM Tris, 1 mM EDTA, adjusted to pH 8.3 by adding acetic acid) containing 12.5 mM $MgCl_2·6H_2O$.

Annealing protocol

For 6HB, the strand mixtures were annealed in a PCR thermos cycler (S1000 Thermal Cycler, Bio-Rad) by the following cooling step:

1. 85 °C 10 min
2. 85 °C→20°C -1 °C / 30 min
3. Hold at 20 °C

Purification of DNA origami

The bare 6HBs were purified from excess staples by polyethylene glycol (PEG) precipitation and redispersion in buffer containing different MgCl2 concentration. Briefly, the 6HBs was mixed in a 1:1 volumetric ratio with PEG precipitation buffer (15%, w/v PEG (Mw: 8000), 505 mM NaCl, 10m MMgCl$_2$, 5 mM Tris, 1 mM EDTA) and centrifuge at 16000 × g for 25 min. The supernatant was discarded and the pellet was re-suspended in 1× TE buffer containing different MgCl$_2$ concentrations. The solution was shaken for 2h at room temperature. The concentration of purified 6HB were measured by nanodrop 2000 spectrophotometer (Thermo Scientific). Even though PEG precipitation is a convenient approach to remove the free staples, but lead to aggregation in some degree, especially for the flat DNA origami structures[1]. In our experiment, we verified the structure integrity and dispersibility of 6HBs with AFM before the silicification.

We designed and assembled 6HB with initiators at different prescribed positions. The folded DNA origamis were purified by agarose gel to remove the excess staple strands. Annealed samples were loaded in the 1% gel and conducted at 70 volts for 2-3 hours in 0.5× TBE with 10 mMMgCl$_2$ and 0.005% (v/v) EtBr in an ice water bath. Then the target bands were excised and placed into a Freeze ’N Squeeze column (Bio-Rad Laboratories, Inc.). The column was stored in -20 °C for 10 min and then centrifuged at 6000 G for 8 min. Gel purified samples were buffer-exchanged into a 1× TdT reaction buffer before TdT-catalyzed enzymatic polymerization reaction using 10 kD Micron centrifugal filters.

Surface-initiated enzymatic synthesis of homopolynucleotide Brushes

Homopolynucleiotide brushes grew on the initiators distributed in different positions of 6HB by surface-initiated TdT-catalyzed enzymatic polymerization. Different numbers of initiators stretched out from different positions of 6HB (e.g., one third at one end with 54 initiators (6HB-9*/18-8T), two thirds at one end with 108 initiators (6HB-18*/27-8T), two ends with 60 initiators (6HB-5*/17/5*-8T), whole body with 162 initiators (6HB-27*-8T)) and adding dTTPs in 1:500 ratio to grow polythymine brush.[2] The initiator concentration (i.e., the number of staples with 3' oligo(dT) overhangs on the origami surface) in these reactions was determined by the concentration of origami multiplied by the number of overhangs per origami. The reaction mixtures contained 1 U/μL TdT in 1× TdT reaction buffer and 500 times dTTP monomers than overhangs 8T concentration. All the enzymatic polymerizations were carried out overnight at 37 °C and characterized by AFM images.

Silicification process

For 6HB without polyT brushes, DNA origami samples were resuspended in 1× TE buffer with different concentrations of MgCl$_2$ (5, 7, 12.5, and 16mM). For 6HB with polyT brushes, after gel purification and TdT reactions, the concentration of MgCl$_2$ was reduced to 5mM in the DNA origami samples. The silicification reactions were carried out in a 30 μL reaction in PCR tubes. A small volume (0.5 μL to 1.5 μL) of 18-72 nmol TMAPS (to achieve intended [6HB]/TMAPS ratios) was added to the DNA origami solution and the final concentration of DNA origami was adjusted to 2 nM by adding 1× TE with 5 mM MgCl$_2$. The mixture was shaken at 400-600 rpm for 20-30 min

at room temperature. Then a small volume (0.5 μL to 1.5 μL) of 13.2-52.8 nmol TEOS (to achieve intended [6HB]/TEOS ratios) was added, and the mixture was shaken for 4 days. TMAPS and TEOS solutions were diluted with methanol when necessary.

Transmission Electron Microscopy (TEM) preparation and Imaging

Prior to imaging, the carbon coated grids are cleaned by glow discharge for 30 s using a PELCO easiGlow system (Ted pella, Inc.). Then 5 μL of a silica-coated sample was adsorbed for 10 minutes onto glow-discharged, carbon-coated TEM grids. The residue is wicked away by filter paper and washed twice by the deionized water. Imaging was performed using a JEOL JEM-1400 operated at 80 kV. The silica-coated DNA origami were observed without staining.

Atomic Force Microscopy preparation and imaging

AFM images were obtained using an SPM Multimode with Digital Instruments Nanoscope V controller (Veeco). 2 μL of purified sample was applied onto the surface of a freshly cleaved mica chip and left for approximately 2 minutes to allow for adsorption. 50 μL of H_2O was then added onto the mica surface and blow away by nitrogen. The AFM tips model used were SCANASYST-AIR and collected images in air (the peak force setpoint: 250 pN).

Simulation methods

To investigate how DNA brushes influence the condensation of TMAPS-TEOS silica precursors on the surface of DNA origami structures (6HBs), we carried out molecular dynamics (MD) simulations of a single brush-functionalized 6HB suspended in an aqueous solution of the silica precursors. In these simulations, we used a coarse-grained (CG) model of the 6HB, brushes, and silica precursors, while the solvent was treated implicitly[3-4]. This allowed us access to the long timescales required for capturing silica condensation while still accounting for the most important structural and dynamical features of the system: the conformational dynamics of single *versus* double-stranded brushes; the geometry and rigid nature of the 6HBs; the confined diffusion motion of the precursors close to the brush-functionalized origamis; and their interactions amongst themselves and with the brushes and origami surface.

CG representation. The 6HB was modeled as a semi-rigid network of CG beads, where the size of each CG bead σ corresponds to a length scale of 2 nm—the effective diameter of a DNA double-helix. The CGs beads were assembled into six linear rows (representing the six DNA helices of the 6HB), each of length of 193σ, and then bundled together into a honeycomb arrangement to mimic the geometry of the 6HB. The resulting rod-like structure approximates the experimentally measured dimensions of the 6HBs (~6 nm in width and ~385 nm in length).

The single-stranded poly-T DNA (ssDNA) strands on the 6HB were each treated as CG bead-chains, 85 σ long to match the 500 nucleotides (~170 nm contour length) long strands in experiments. As depicted in **Figure S17**, the chains were attached to the 6HB following the design of 6HB-5*/17/5*, where each row of 6HB CG beads was grafted with 10 CG poly-T chains, with 5 chains (with 6σ spacing between attachment points) placed at one end and the remaining 5

chains at the other end (also with 6σ spacing). Also consistent with the experimental system, alternating rows of attachment points were shifted by 2σ relative to each other. In total, there were 60 poly-T chains on the 6HB. In the case of double-stranded brushes (single stranded DNA with closely spaced hairpins; dsDNA), additional side chains of length 2σ (i.e., 2 CG beads) and spacing 2σ representing the hairpins were added along the chain.

Lastly, the small clusters of silica precursors typically formed in solution before depositing onto the DNA structures were phenomenologically represented as CG beads of size 1σ. While the size of these clusters ("primary particles") is variable and not fully known, we assumed for convenience a uniform size of 2 nm roughly corresponding to 2 to 6 molecules[5-6]. More detailed modeling of these precursors that correctly depicts their clustering behavior with atomistic details is not compatible with the coarse resolution of our model and would be far more computationally expensive. For convenience, we assumed all CG beads, irrespective of their chemical constituents, to have the same mass of $m =$ 4,000 amu.

CG intramolecular interactions. The chains and 6HB were treated as bead-spring models, and adjacent beads denoting *bonded segments* interacted with each other through a combination of finitely extensible nonlinear elastic (FENE) spring and Weeks-Chandler-Anderson (WCA) potentials[7]. The FENE spring potential, which ensures that bonded segments do not stretch beyond a cutoff distance, is given by:

$$U_{\mathrm{FENE}}(r; k, R_0) = -\frac{k}{2} R_0^2 \ln\left[1 - \left(\frac{r}{R_0}\right)^2\right] \qquad (1)$$

where r is the separation distance between segments, $k = 30\varepsilon_0/\sigma^2$ is the spring constant, ε_0 determines the energy scale of our system taken to be $1\,k_{\mathrm{B}}T$ (k_{B} is Boltzmann constant), and $R_0 = 1.5\,\sigma$ is the maximum possible length of the spring. The WCA potential, a short-range purely repulsive potential that models excluded-volume interactions between the bonded segments, is given by:

$$U_{\mathrm{WCA}} = \begin{cases} 4\varepsilon\left[\left(\frac{\sigma}{r}\right)^{12} - \left(\frac{\sigma}{r}\right)^{6}\right] + \varepsilon & r < 2^{1/6}\sigma \\ 0 & r \geq 2^{1/6}\sigma \end{cases} \qquad (2)$$

where $\varepsilon = \varepsilon_0$. The same FENE-WCA potential was also used for tethering the terminal CG beads of the single- and double-stranded chains to appropriate surface CG beads of the 6HB.

To account for the higher stiffness of the double-stranded DNA chains and the 6HB rods, we implemented a harmonic bending potential on all triplets of adjacent CG beads in these two components. Note that in the case of the 6HB, there are two types of such triplets, those connecting beads along the rows and those in the circumferential direction. This potential can be expressed as

$$U_{\text{bending}} = \frac{K}{2}(\theta - \theta_0)^2 \qquad (3)$$

Where $K = k_{\mathrm{B}}Tl_{\mathrm{p}}/\sigma = 25\varepsilon_0$ is the bending constant that depends on the persistence length $l_{\mathrm{p}} = 50\ \mathrm{nm}$ of dsDNA and $\theta_0 = 180°$ is the equilibrium bending angle[8], except for the triplets

connecting beads along the circumferential direction in the 6HB, where we used $\theta_0 = 120°$ to maintain the honeycomb topology of 6HB. We did not apply any bending potential to the single-stranded chains as their persistence length is much smaller than σ, and thus they can be modeled as a freely jointed chain.

CG intermolecular interactions. Both the DNA brushes and the 6HB are strongly negatively charged and the silica precursor clusters are strongly positively charged. To model the repulsive or attractive electrostatic interactions between these charged components, we assigned effective charges to the CG beads making up each component and treated these interactions using a Debye-Hückel (DH) potential[3]. The DH potential accounts for the screening effect caused by the presence of counterions (Mg^{2+} and Cl^{-} ions) with an exponentially damping factor:

$$U_{\mathrm{DH}} = \begin{cases} \frac{q_i q_j}{4\pi\epsilon_0 \epsilon r}\exp(-\kappa r) & r < r_{\mathrm{ce}} \\ 0 & r \geq r_{\mathrm{ce}} \end{cases} \quad (4)$$

where κ is the inverse Debye screening length, ϵ is the dielectric constant of the solvent, ϵ_0 is the permittivity of vacuum, q_i and q_j are charges on the interacting CG beads i and j, and r_{ce} is a cutoff distance imposed to save computational cost. We set $\epsilon = 78$ based on the dielectric constant of pure water at room temperature. Using $[MgCl_2]$ = 5 mM used in experiments (effective ionic strength $I = 3[\mathrm{Mg}^{2+}] = 15\mathrm{mM}$) and e as the elementary charge in the Debye-Hückel model, we obtain a large Debye length of $\kappa^{-1} = \sqrt{2e^2 I/\epsilon\epsilon_0 k_{\mathrm{B}} T} \approx 1.25\sigma$, i.e., 2.5 nm, which clearly does not reflect the much stronger screening power of divalent ions like Mg^{2+}. To this end, we explored various smaller values of κ^{-1} in the range 1.0 to 2.5 nm. Since the DH potential becomes negligible at distances much larger than the Debye length, we chose a value of $r_{\mathrm{ce}} = 6.5\sigma$ as the cutoff for this potential. Based on the spacing between charged phosphate groups (the average distance between base pairs or bases), which is ~0.34 nm for dsDNA and ~0.63 nm for ssDNA [9], we assigned charges of $11.8e$ per bead of the 6HB (q_{6HB}) as well as the double-stranded brushes (q_{dsDNA}), and $3.2e$ per bead for single-stranded brushes (q_{ssDNA}). However, since the precise size and charge on the silica clusters is not known, we explored several different values in the range q_{silica} in the range $2e$ to $6e$.

In addition to electrostatic interactions considered above, silica precursor interactions require special care as they can undergo self-polymerization, indicating the presence of attractive interactions that lead their condensation. However, modeling such polymerization with the correct atomistic details is unachievable in our CG model. Hence, we approximated the attraction leading to condensation using an attractive shift-corrected LJ potential given by

$$U_{\mathrm{LJ}} = \begin{cases} 4\varepsilon_{\mathrm{silica}}\left[\left(\frac{\sigma}{r}\right)^{12} - \left(\frac{\sigma}{r}\right)^{6} - \left(\frac{\sigma}{r_{\mathrm{ca}}}\right)^{12} + \left(\frac{\sigma}{r_{\mathrm{ca}}}\right)^{6}\right] & r < r_{\mathrm{ca}} \\ 0 & r \geq r_{\mathrm{ca}} \end{cases} \quad (5)$$

with a standard cutoff distance of $r_{\mathrm{ca}} = 2.5\sigma$. Like the charge on silica precursors, the strength of this attraction is also not known, so we explored different values of $\varepsilon_{\mathrm{silica}}$ in the range ε_0 to $3\varepsilon_0$.

MD simulations. The LAMMPS program was used for carrying out MD simulations[10]. Each simulation included 3,375 clusters of silica precursors surrounding a single brush-functionalized 6HB in a periodic simulation box of dimensions $400\sigma \times 400\sigma \times 400\sigma$, sufficiently large to prevent the 6HB from interacting with its periodic image. The simulated silica precursor concentration was roughly 15 times larger than the experimental value to enable the full condensation process to be captured within reasonable computational times. All simulations were carried out in the canonical (NVT) ensemble at a fixed temperature of 1.0 $\varepsilon_0/k_{\mathrm{B}}$ corresponding to 298 K in real units. A velocity-Verlet algorithm with a timestep of 0.002τ and a Langevin thermostat with a damping constant of $1.0\,\tau^{-1}$ were used for integrating the equations of motion and controlling temperature, where $\tau = (m\sigma^2/\varepsilon_0)^{1/2}$ is the time unit. The system was subjected to equilibration for 0.6 million timesteps to ensure proper dispersion of the precursors throughout the simulation box, and we temporarily set a high damping constant of $100\ \tau^{-1}$ to ensure fast dispersion. To prevent the precursors from condensing during this equilibration stage, their electrostatic and LJ interactions were disabled, whereby the precursor beads interacted with other beads including themselves only via excluded volume interactions. During the production stage, the electrostatic and LJ interactions were turned back on, and the simulations were performed for an additional 27.6 million timestep. We collected three independent simulation trajectories to ensure reliability of our results and obtain better statistics. We used OVITO to visualize the simulation trajectories[11] . We found that $q_{\text{silica}} = 2e$, $\varepsilon_{\text{silica}} = \varepsilon_0$, and $\kappa^{-1} = 1\sigma$ best reproduced the experimentally observed condensation behavior of silica on the 6HBs. Thus, all subsequent analyses are based on this parameter set.

Silica condensation density analysis. A method based on the distance of a silica precursor from the outer surface beads of the 6HB was used for determining their condensation state (adsorbed if the precursor is within a cutoff distance of 2σ from any 6HB bead and in solution if farther away) and for classifying them into two groups (adsorbed in the bare region of the 6HB versus those adsorbed in the brush-functionalized regions). The amount of silica condensation was quantified in terms of the number density—dividing the accumulated number of silica precursors in the two regions by the length of the region (the length of the grafted region is $L_{\text{Grafted}} = 58\,\sigma$, and the length of the bare region is $L_{\text{Bare}} = 135\sigma$). The number density was averaged over time intervals of 2000τ, and a standard error of the mean is also reported.

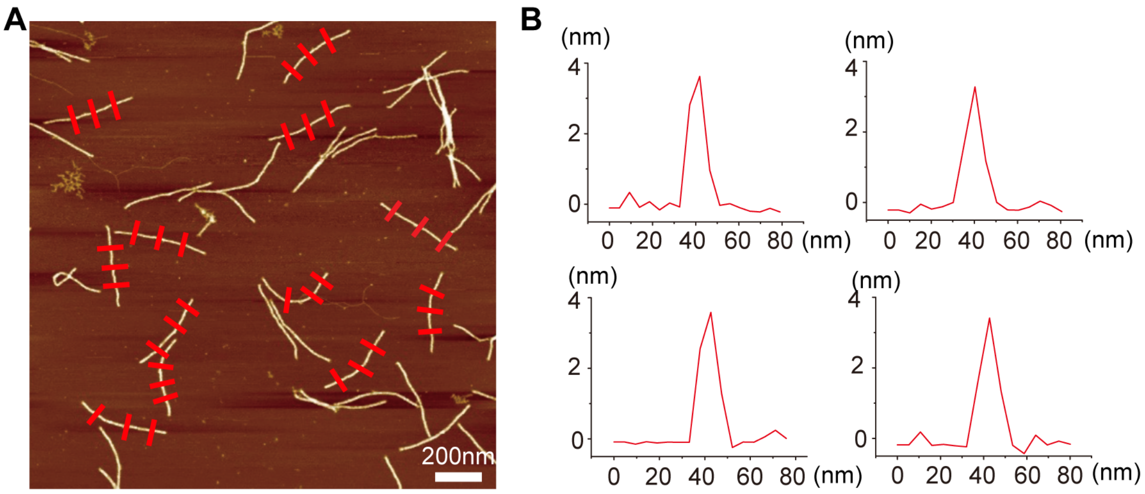

Figure S1. Height measurement of 6HB after silicification. (A) AFM of 6HB after silicification under 5 mM Mg^{2+} and height measured from AFM images **(B)**. As the Figure S1A shows, we choose 30 samples randomly in the picture, and measured the heights at three different positions on one sample, totally collecting 90 heights. The means and standard deviations of 90 heights of different samples were used to obtain Figure 1C and 1E.

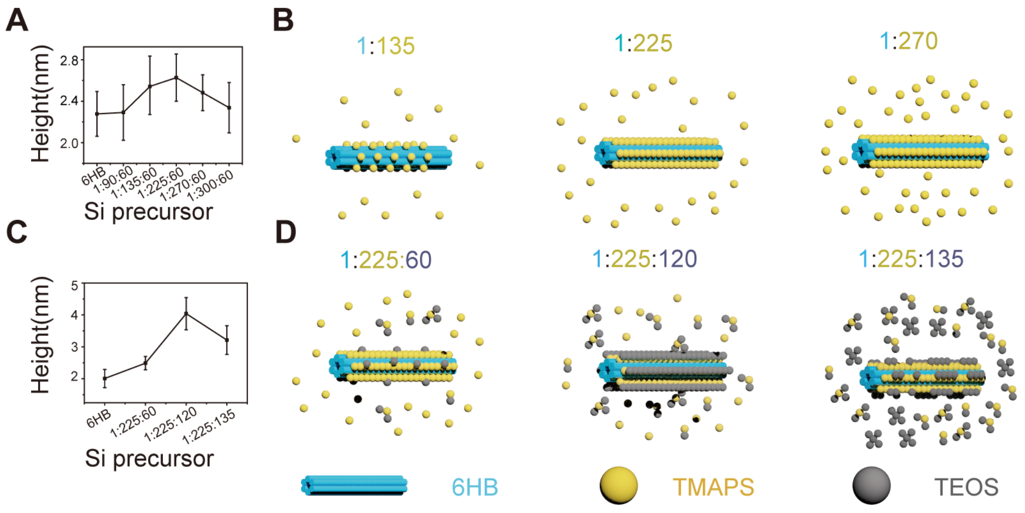

Figure S2. Analysis of silicification process. (A) Height changes of 6HB as TMAPs increased. **(B)** Schematic diagram of distribution of TMAPs on 6HB and in solution as TMAPs increased. All TEOS are added at a 1:60 ratio. **(C)** Height changes of 6HB as TEOS increased. **(D)** Schematic diagram of distribution of TEOS on 6HB and in solution as TEOS increased. All TMAPs are added in a 1:225 ratio. 1:225:60 means the molar ratio of phosphate groups in 6HB to TMAPS reagents to TEOS reagent.

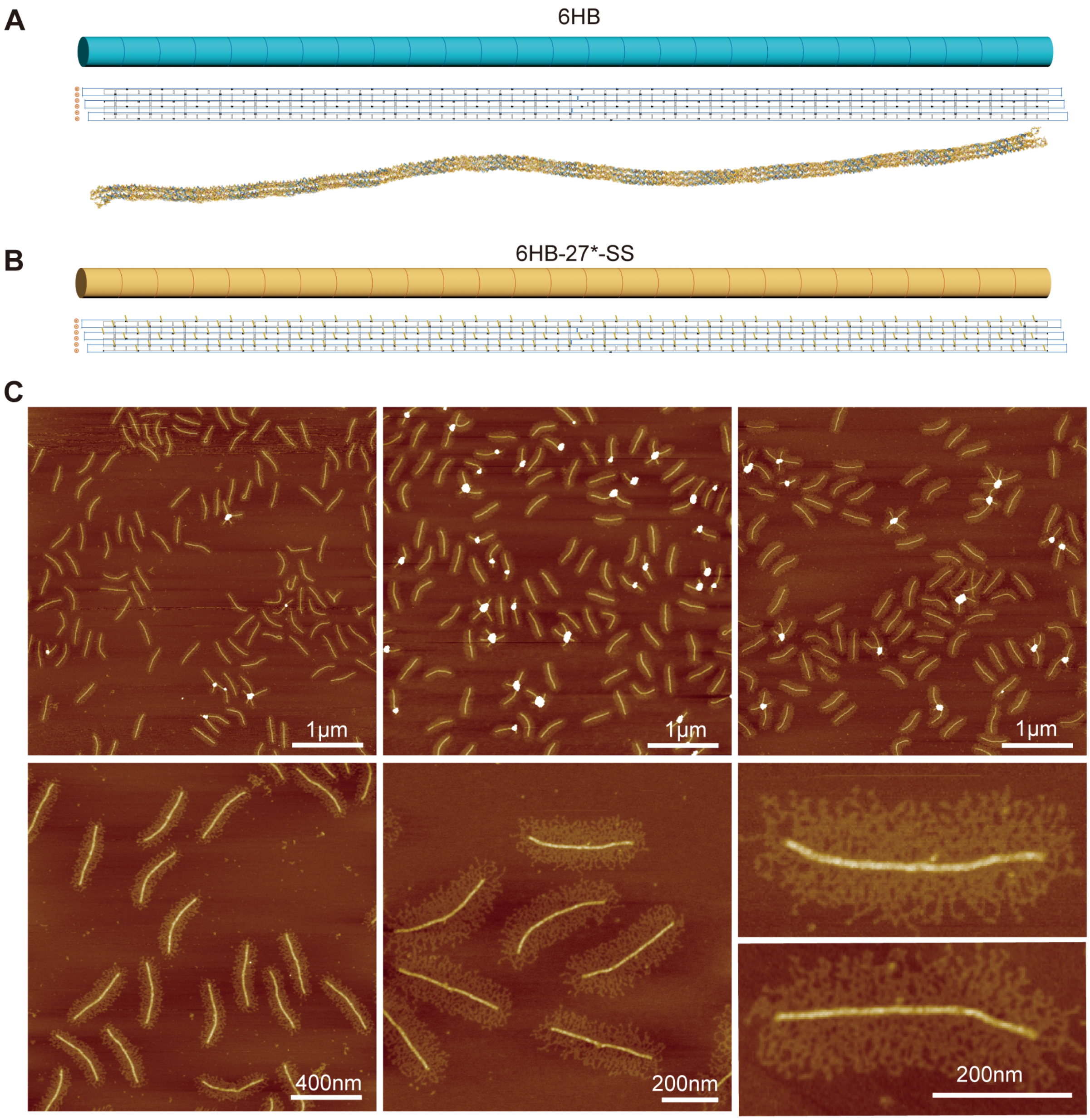

Figure S3. Design and characterization of 6HB without and with polyT modification. (A) Schematic diagram of 6HB and its OXDNA model, caDNAno design. **(B)** The model of 6HB-27*-SS and the caDNAno design with polyT growth positions. **(C)** AFM images of 6HB-27*-SS.

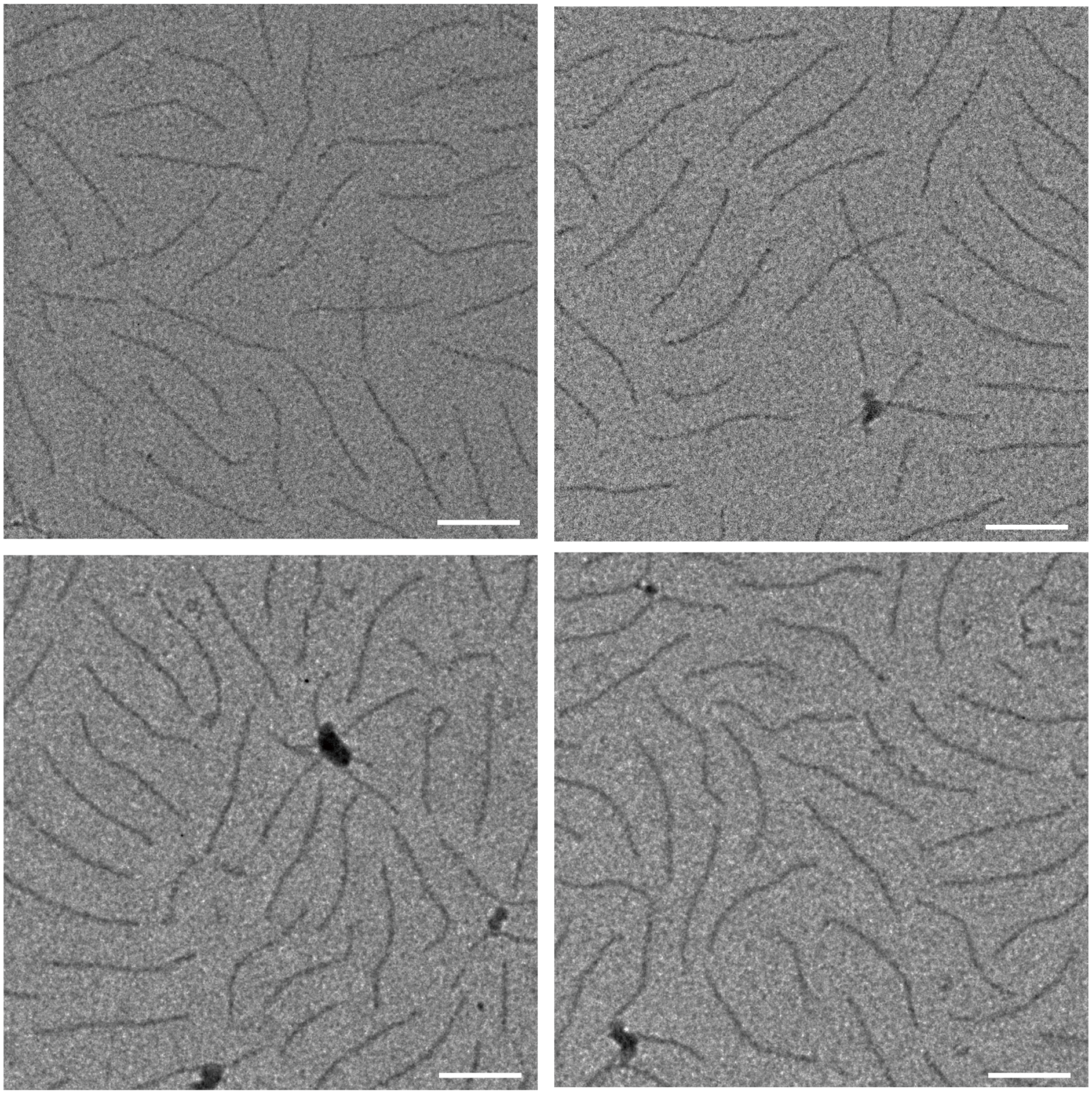

Figure S4. TEM characterization of 6HB-27*-SS. TEM images of 6HB-27*-SS after silicification for 1 day without staining. Scale bars are 200 nm.

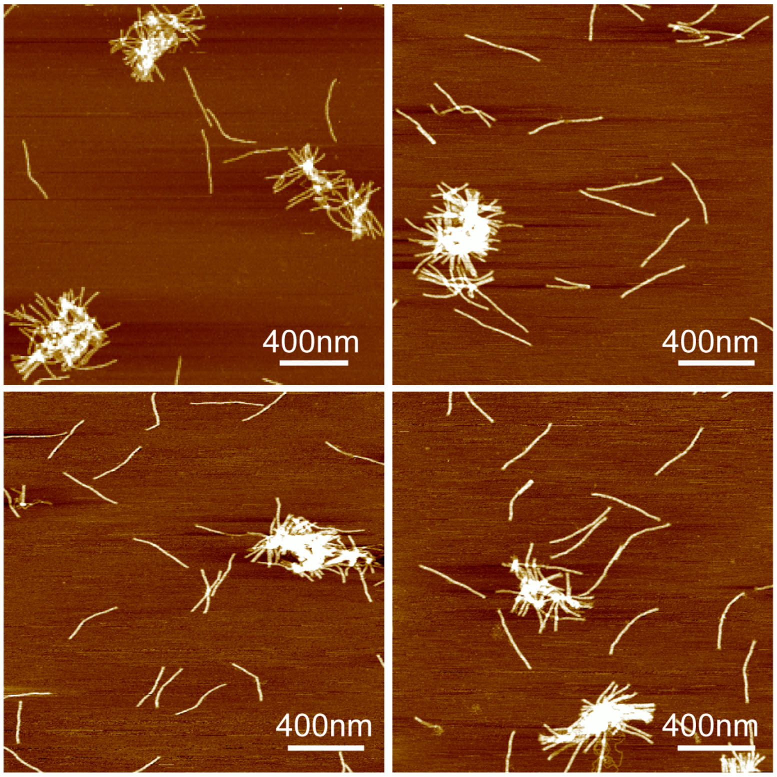

Figure S5. AFM images of 6HB after silicification over two days under 35 °C. Significant amount of aggregation were observed.

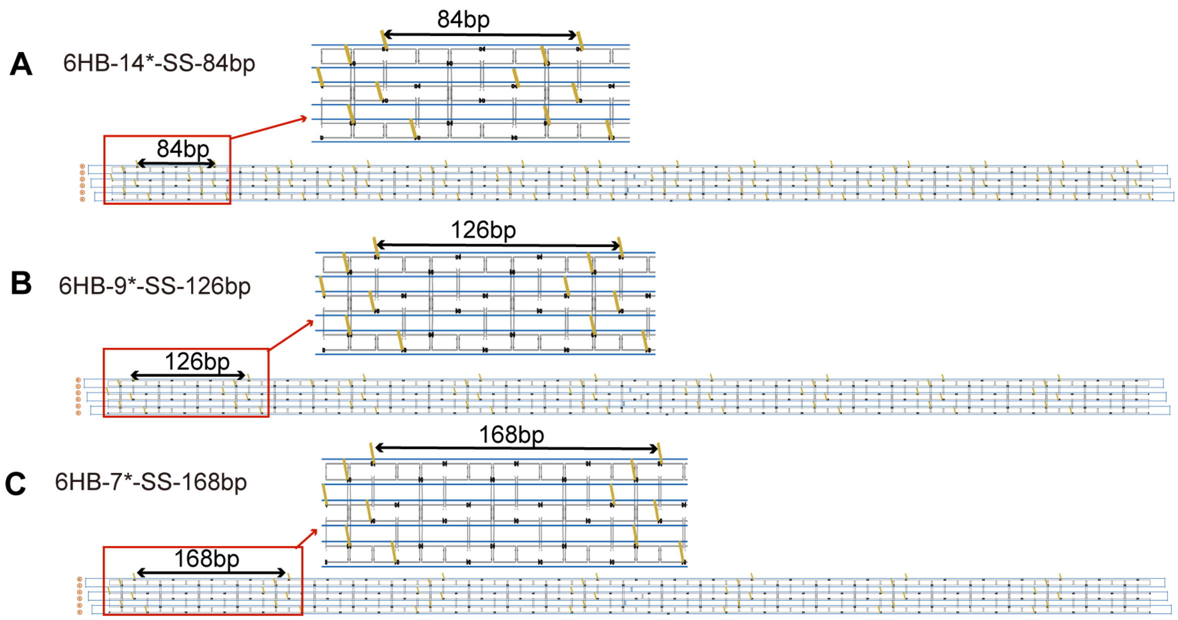

Figure S6. Schematic diagram of 6HB with different density polyT. The distances of polyT brushes were 84 bp (**A**), 126 bp (**B**) and 168 bp (**C**), respectively.

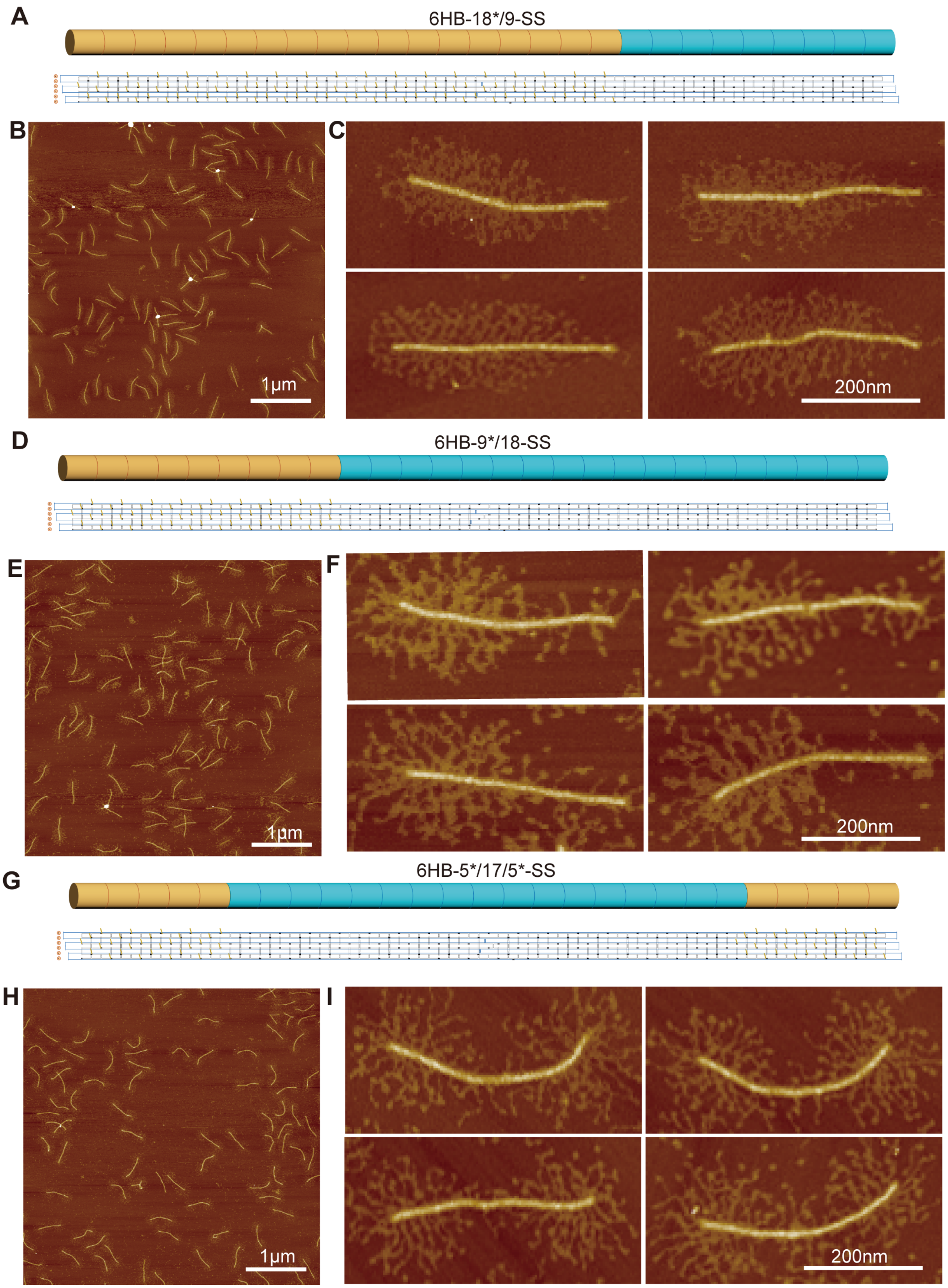

Figure S7. Design and characterization of three 6HB structures with partially modified polyT brushes. Model, design and AFM images of 6HB-18*/9-SS (**A, B, C**), 6HB-9*/18-SS (**D, E, F**), 6HB-5*/17/5*-SS (**G, H, I**).

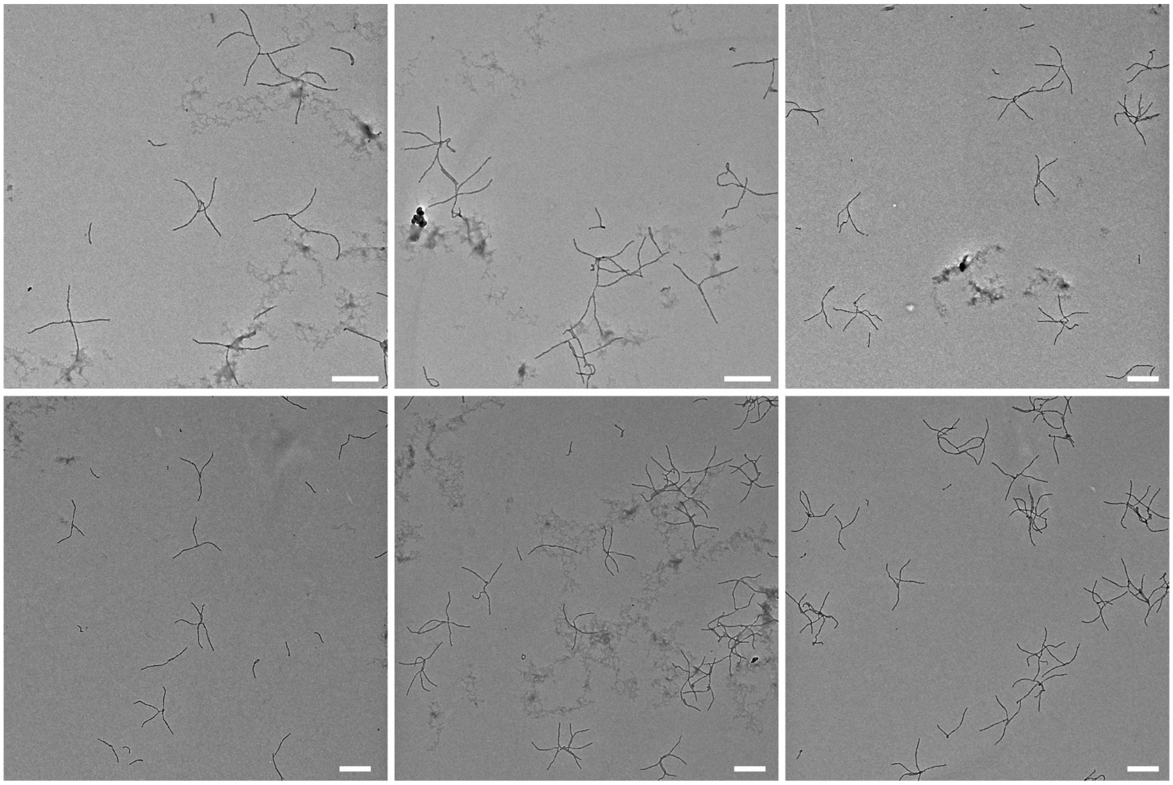

Figure S8. TEM images of 6HB-18*/9-SS after silicification reaction. $C_{DNA\ origami}$ = 2 nM, C_{TMAPS} = 2 mM, C_{TEOS} = 2.64 mM, growth time is 4 days. Scale bar is 400 nm.

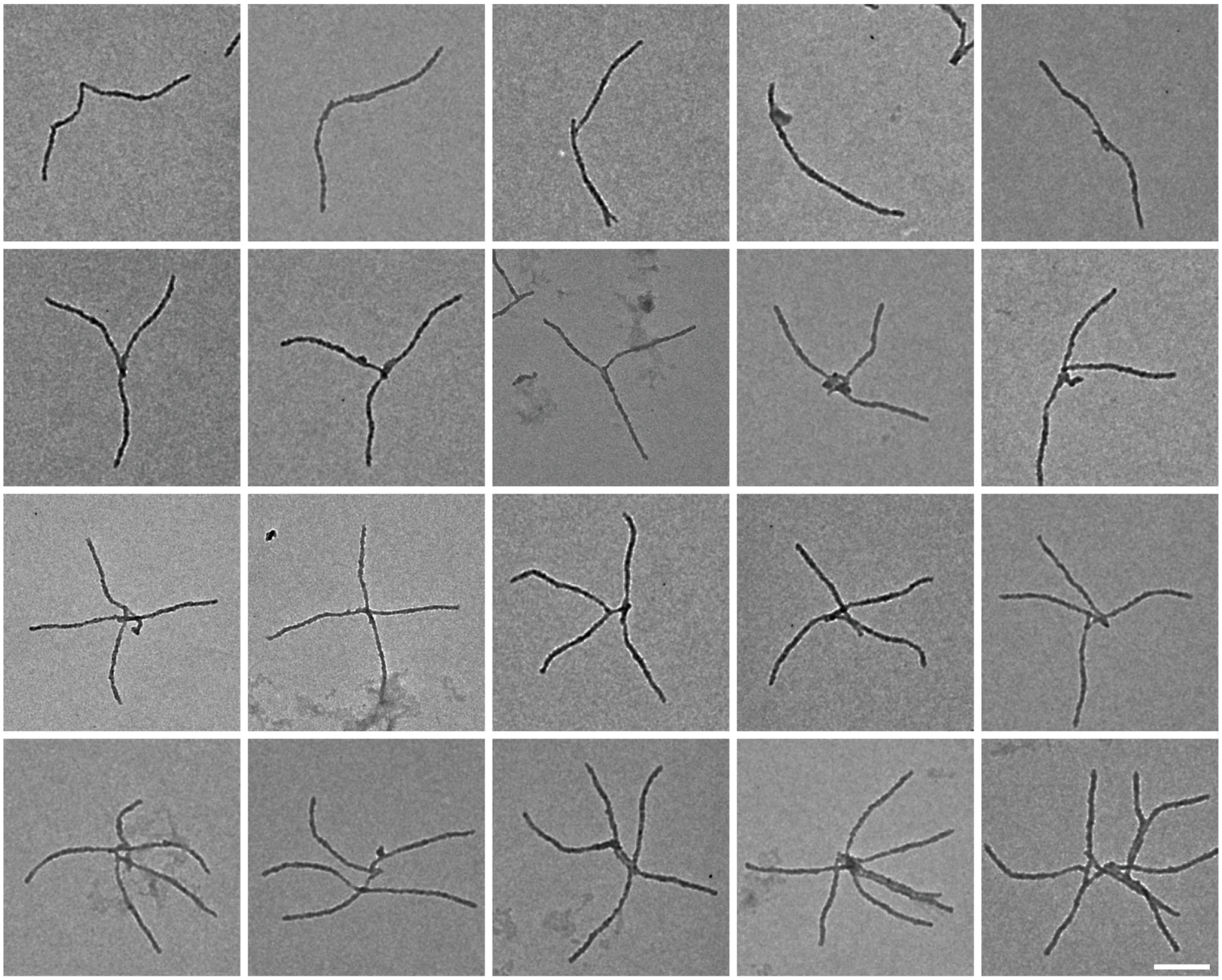

Figure S9. Zoom in TEM images of 6HB-18*/9-SS after silicification reaction. $C_{DNA\ origami}$ = 2 nM, C_{TMAPS} = 2 mM, C_{TEOS} = 2.64 mM, growth time = 4 days. Scale bar is 200 nm.

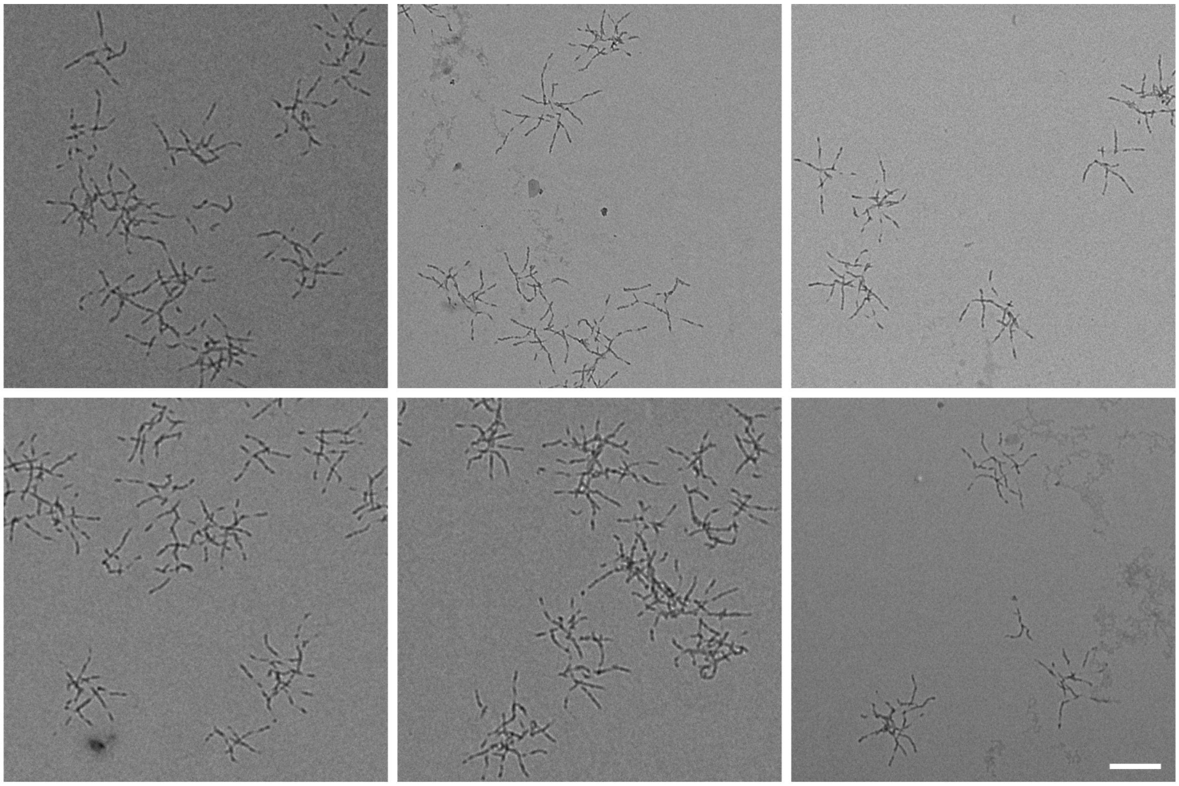

Figure S10. TEM images of 6HB-9*/18-SS after silicification reaction. $C_{DNA\ origami}$ = 2 nM, C_{TMAPS}=2 mM, C_{TEOS} = 1.8 mM, growth time = 4 days. Scale bar is 400 nm.

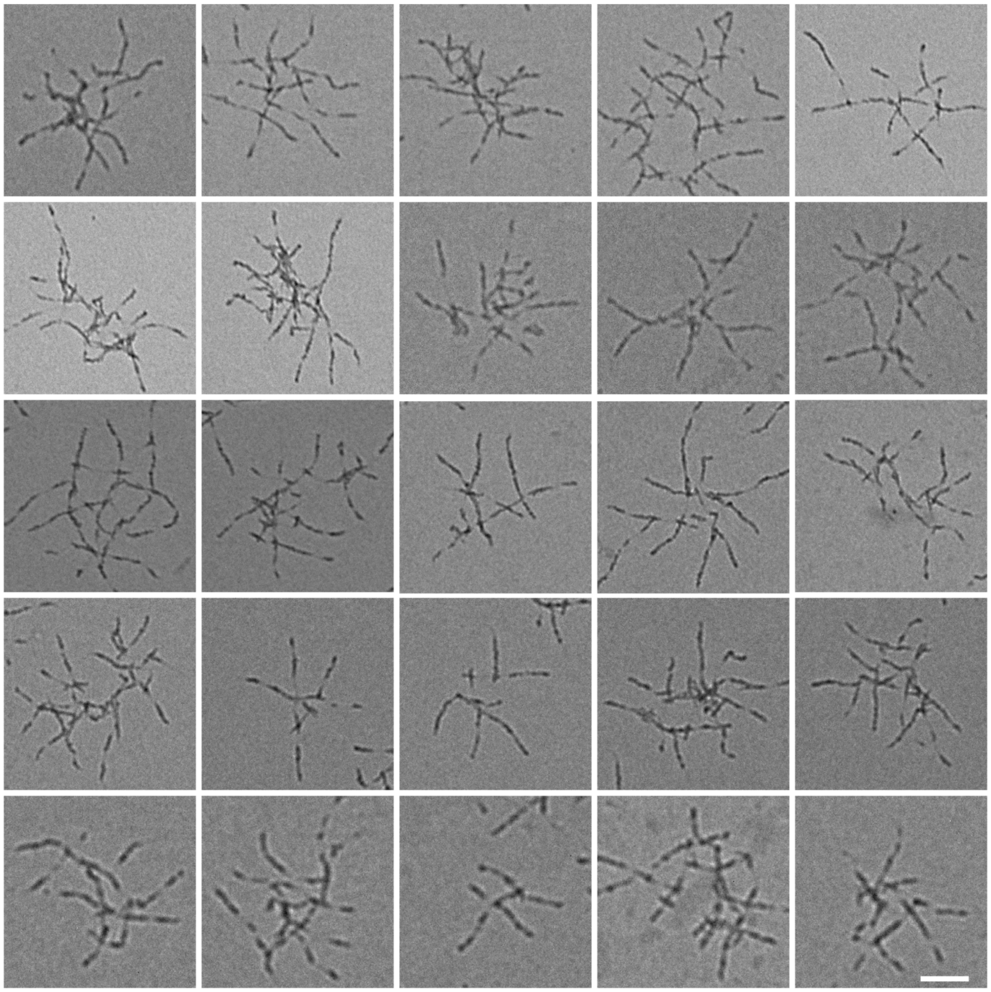

Figure S11. Zoom in TEM images of 6HB-9*/18-SS after silicification reaction. $C_{DNA\ origami}$ = 2 nM, C_{TMAPS} = 2 mM, C_{TEOS} = 1.8 mM, growth time = 4 days. Scale bar is 200 nm.

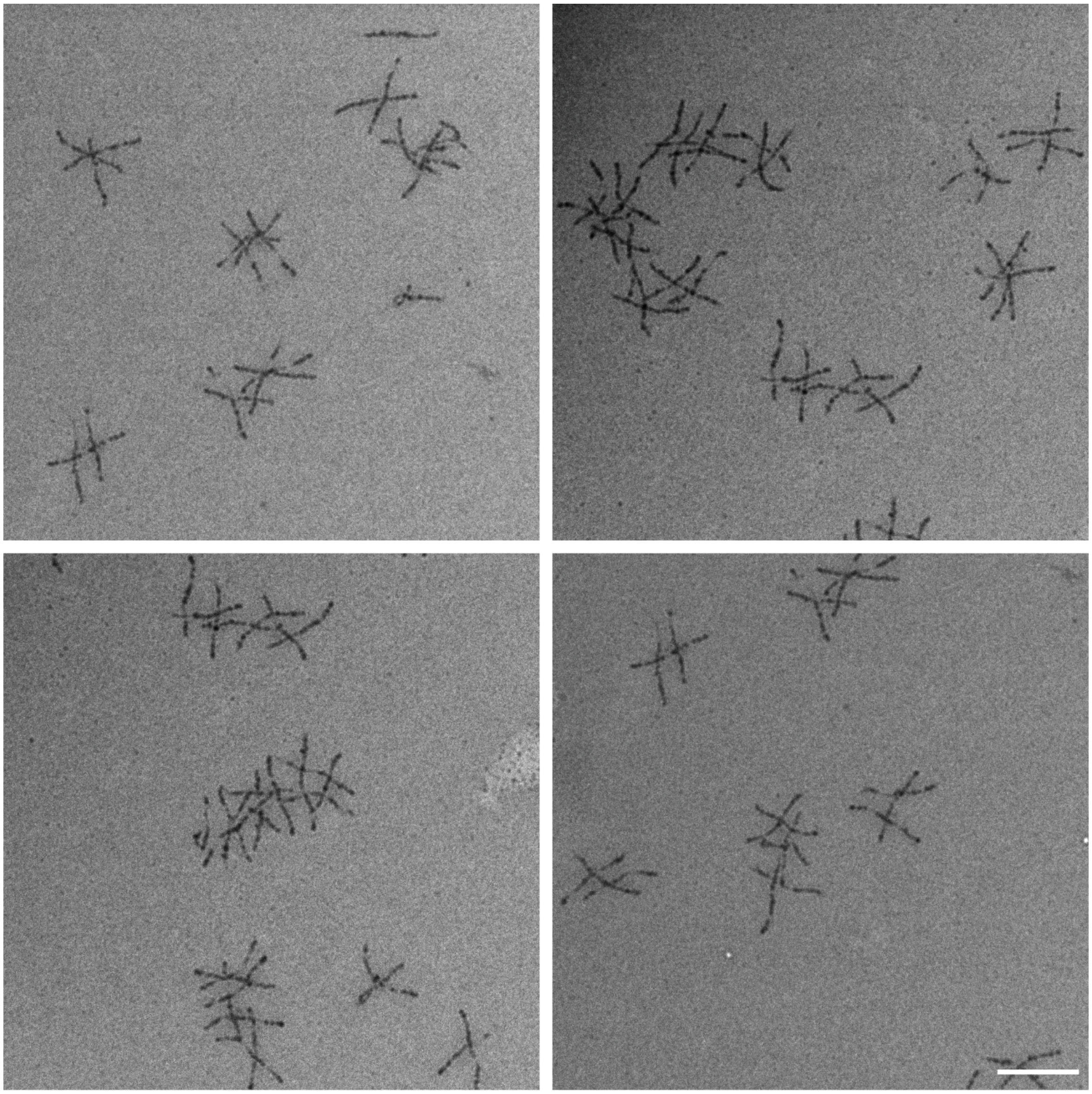

Figure S12. TEM images of 6HB-5*/17/5*-SS after silicification reaction. $C_{DNA\ origami}$ = 2 nM, C_{TMAPS} = 2 mM, C_{TEOS} = 2 mM, growth time = 4 days. Scale bar is 400 nm.

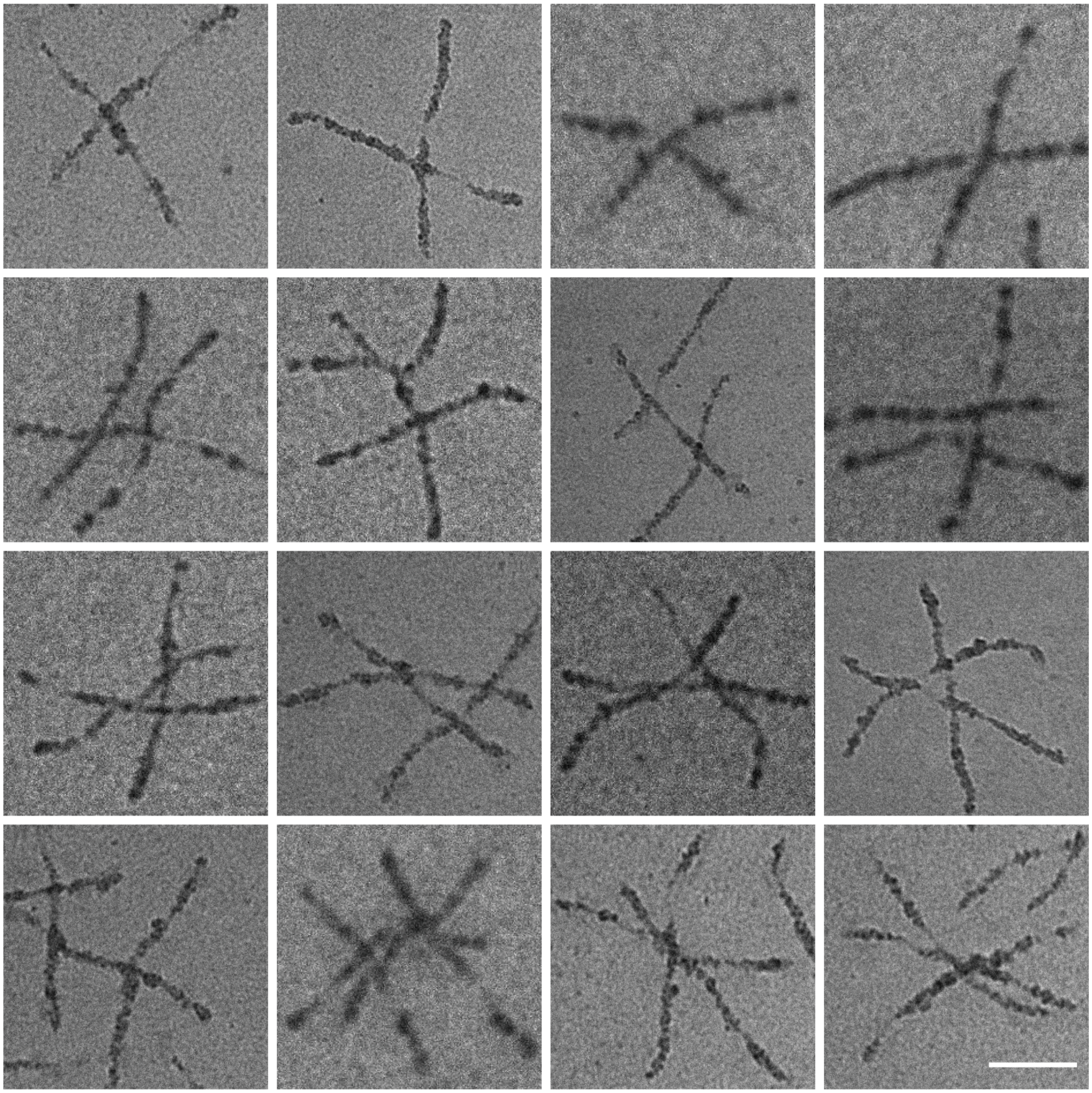

Figure S13. Zoom in TEM images of 6HB-5*/17/5*-SS after silica coating. $C_{DNA\ origami}$ = 2 nM, C_{TMAPS} = 2 mM, C_{TEOS} = 2 mM, growth time = 4 days. Scale bar is 200 nm.

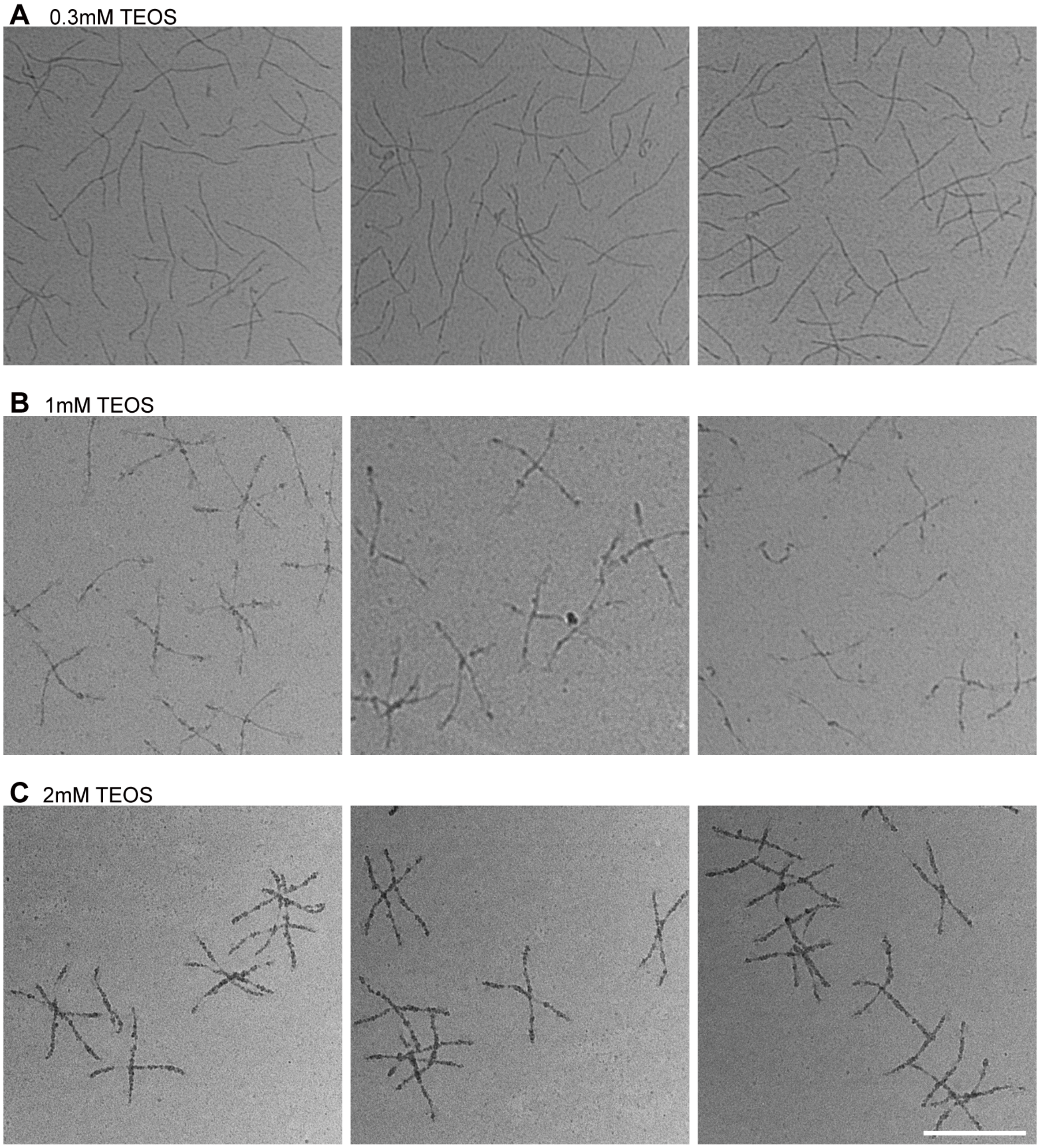

Figure S14. TEM images of 6HB-5*/17/5*-SS after adding different silica precursors. (A) 0.3 mM TEOS, **(B)** 1 mM TEOS, **(C)** 2 mM TEOS. $C_{DNA\ origami}$ = 2 nM, C_{TMAPS} = 2 mM, growth time is 4 days. Scale bar is 400 nm.

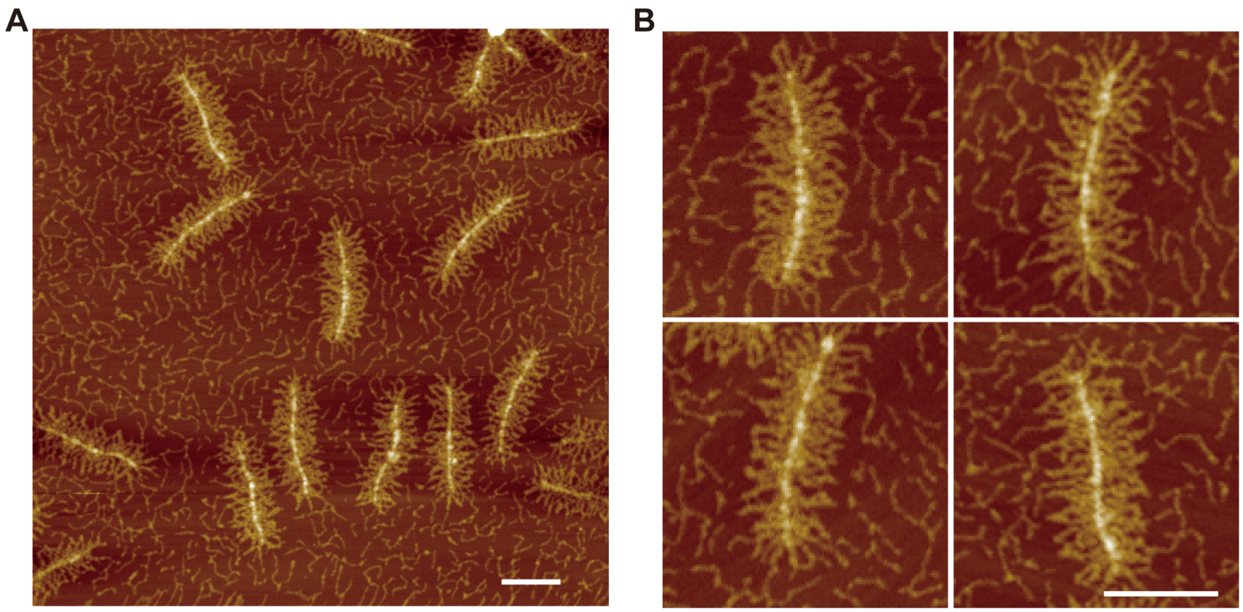

Figure S15. Characterization of 6HB modified with double-stranded brushes. (**A, B**) AFM images of 6HB-27*-DS. Scale bars are 200 nm.

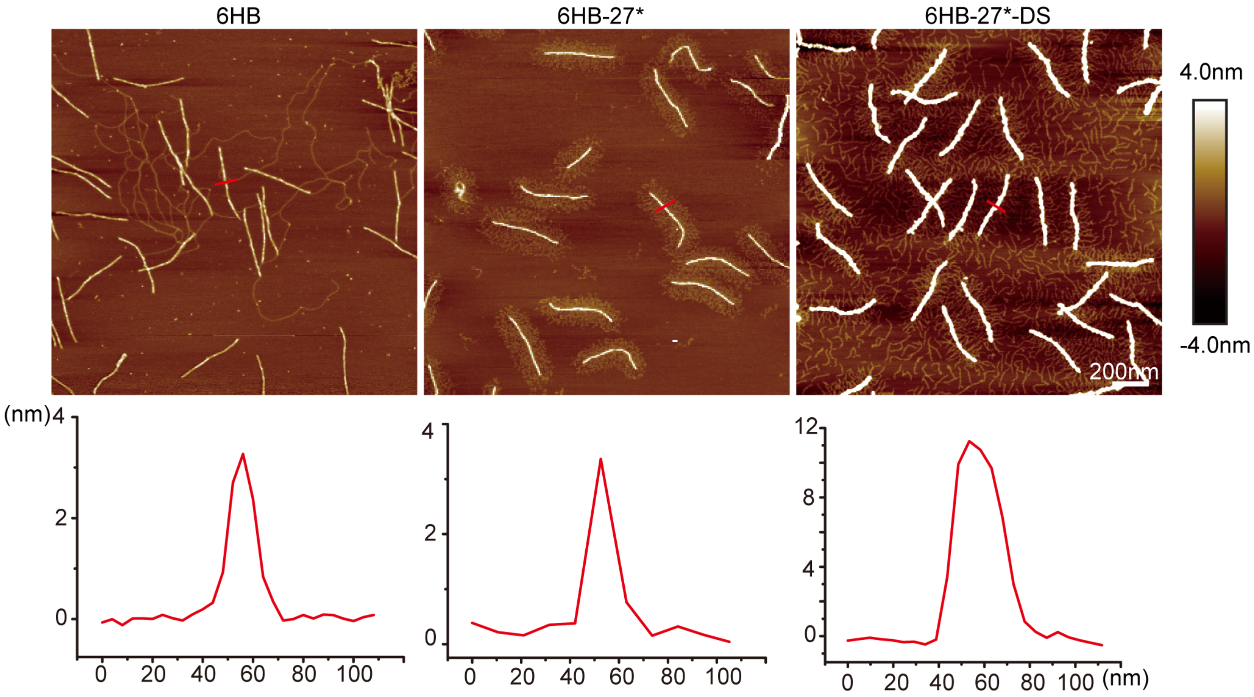

Figure S16. Effect of single-stranded and double-stranded polyT brushes on silicification. $C_{DNA\ origami}$ = 2 nM, C_{TMAPS} = 2 mM, C_{TEOS} = 1.1 mM, growth time = 3 days.

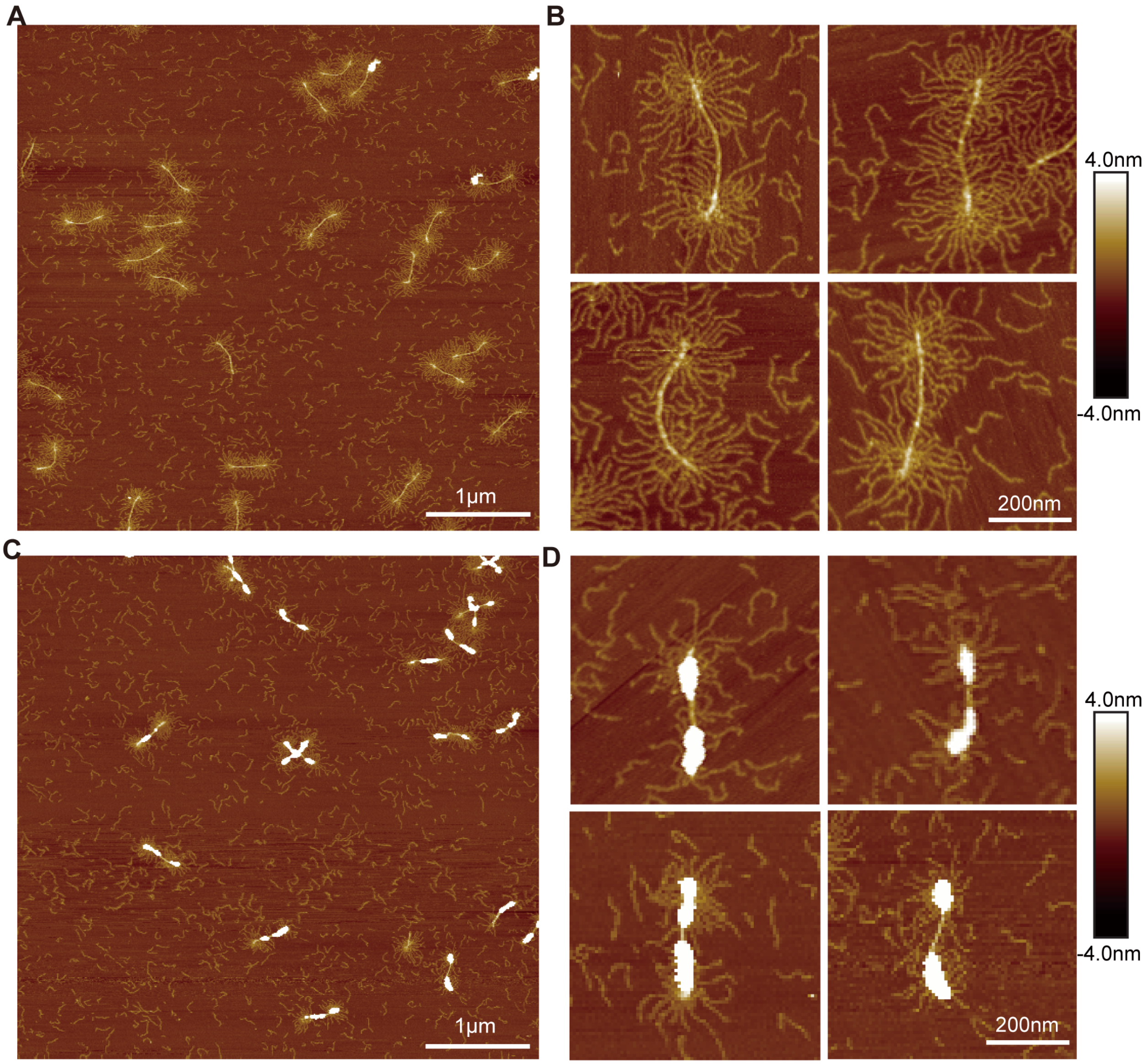

Figure S17. Selective silicification on DNA origami by introducing hairpin DNA at prescribed two ends of 6HB. AFM images of 6HB-5*/17/5*-DS before (**A, B**) and after (**C, D**) silicification.

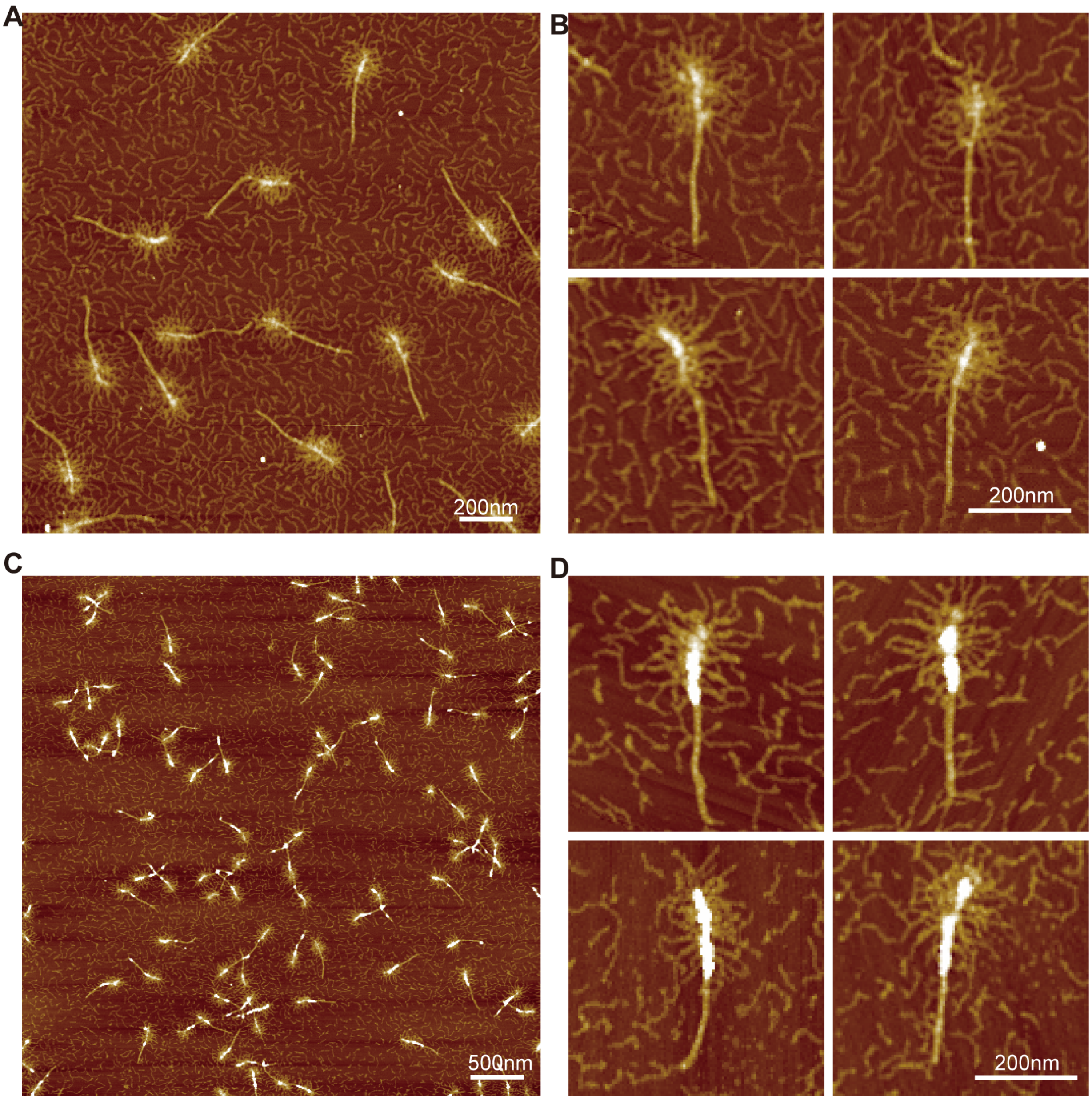

Figure S18. Selective silicification on DNA origami by introducing hairpin DNA at prescribed one end of 6HB. AFM images of 6HB-9*/18-DS before (**A, B**) and after (**C, D**) silicification.

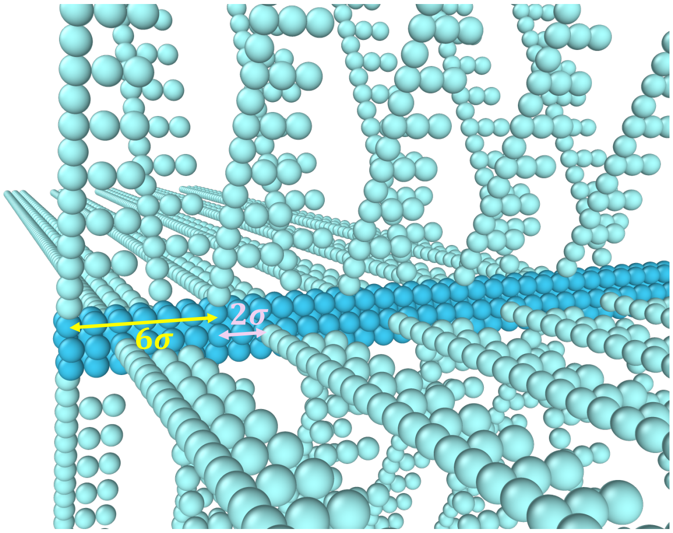

Figure S19. Coarse-grained (CG) representation of a 6HB functionalized with double-stranded DNA brushes. Only one end of the 6HB is shown. Each row of 6HB CG beads was grafted with ten CG polyT chains containing double-stranded hairpins. Five chains were attached at one end of the 6HB with a spacing of 6σ between them, and the remaining five chains were positioned at the other end, also with a spacing of 6σ. The attachment points in alternating rows were shifted by 2σ relative to each other. In total, there are 60 chains on the 6HB.

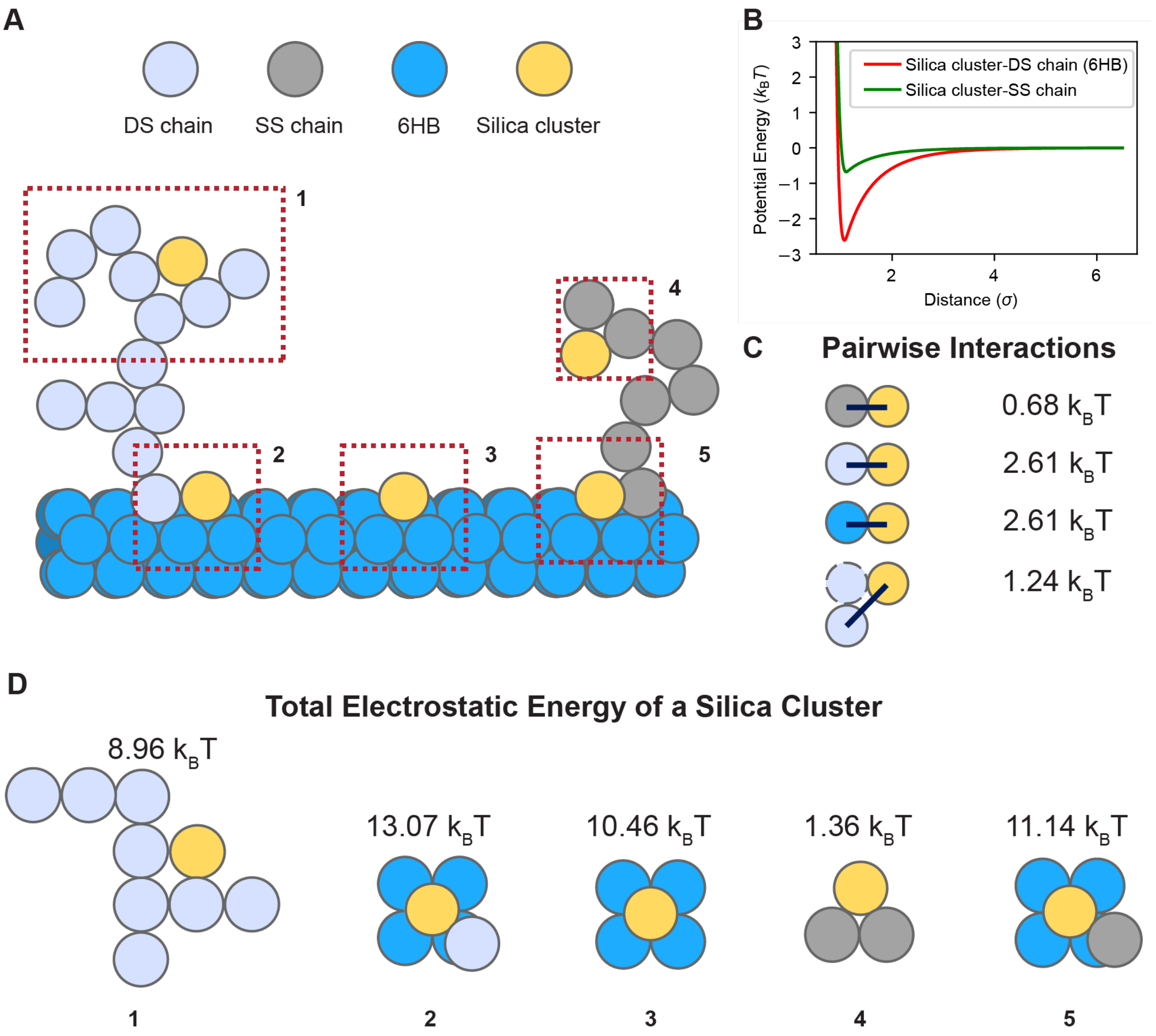

Figure S20. Energetic gain of silica precursor clusters binding to brush-functionalized 6HB. (**A**) Single silica precursor cluster adsorbed at five different sites labeled 1 through 5 (1: double-stranded DNA chain; 2: 6HB surface close to double-stranded DNA; 3: bare portion of the 6HB surface; 4: single-stranded DNA chain; 5: 6HB surface close to single-stranded DNA). (**B**) Electrostatic potentials ($U_{\mathrm{DH}} + U_{\mathrm{WCA}}$) for describing the pairwise interactions between a silica cluster and a single-stranded or double-stranded DNA bead. (**C**) Estimated strength of pairwise interactions based on given interparticle distance. (**D**) Net electrostatic interaction energies of a single silica cluster adsorbing at those five different sites.

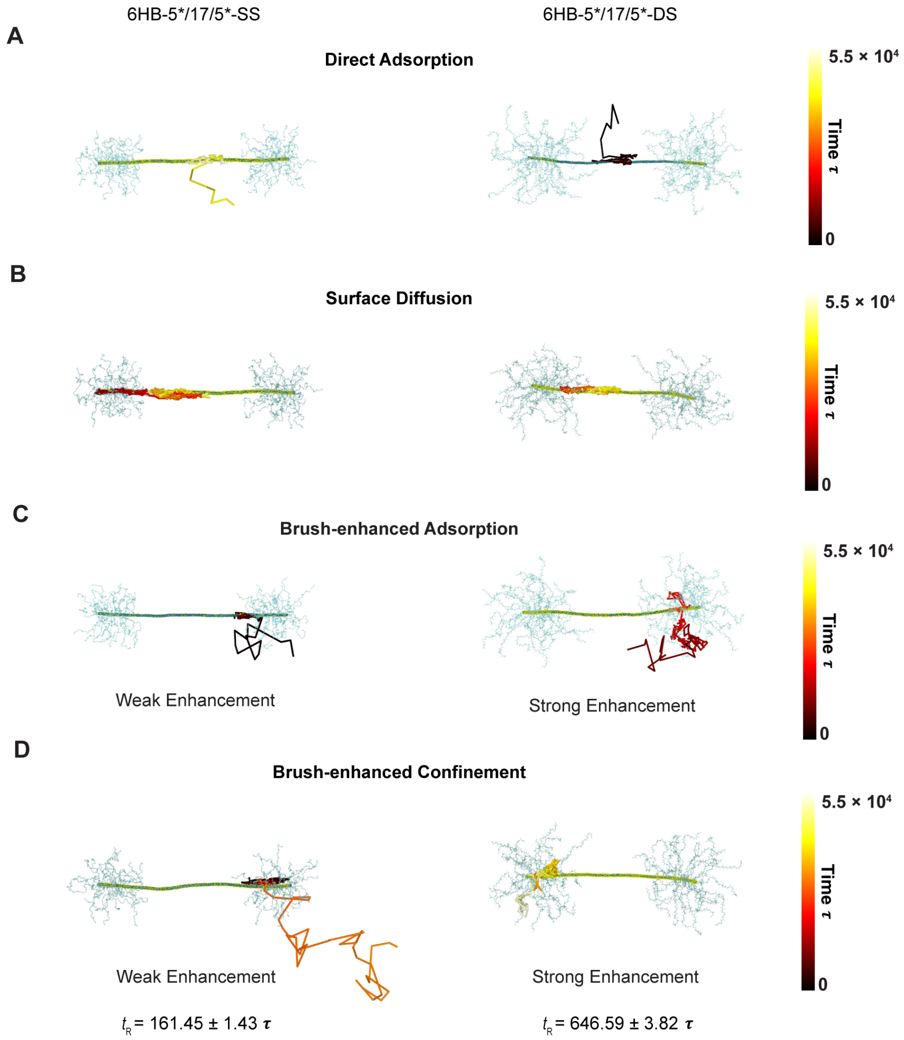

Figure S21. Representative simulation trajectories demonstrating their different dynamic characteristics along with mean residence time analysis. We identified four types of dynamics in the trajectories of a single silica precursor interacting with 6HB-5*/17/5*-SS (left column) and 6HB-5*/17/5*-DS (right column): (**A**) direct adsorption, where a precursor directly adsorbs onto the 6HB without brushes influencing its kinetics, (**B**) surface diffusion, where a precursor adsorbed on the brush-grafted region of 6HB transports onto the bare portion of the 6HB; (**C**) brush-enhanced adsorption, where a precursor's adsorption kinetics is accelerated by the presence of brushes, and (**D**) brush-enhanced confinement, where a precursor can be retained by brushes, preventing its escape from the 6HB. The brush enhancement in adsorption and confinement is more significant in the case of 6HB-5*/17/5*-DS, where the precursor's trajectories are heavily influenced by the presence of double-stranded brushes, whereas for 6HB-5*/17/5*-SS, the single-stranded brushes cannot prevent a precursor from escaping. To understand how the brush enhancement differs for single- and double-stranded brushes, we calculated the mean residence time of precursors t_R within brushes, which is how long a silica cluster stays within the brush region by measuring the time it stays as a neighbor of brush beads until it is not a neighbor of any brush bead, or it becomes a neighbor of a 6HB bead. In this analysis, a neighbor is defined as being within two Debye lengths of a given bead. The mean and the standard error of the computed residence time are reported at the bottom.

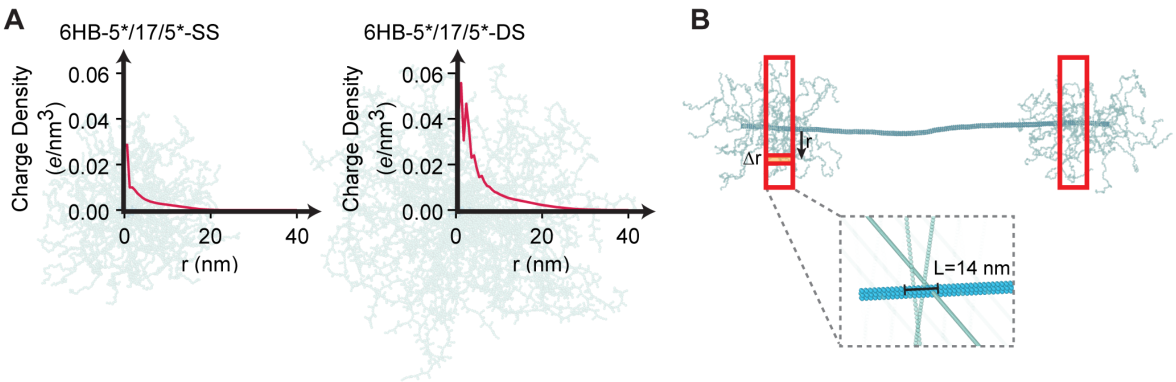

Figure S22. Brush charge density analysis. (**A**) Charge density provided by brushes as a function of distance r from the surface of 6HB-5*/17/5*-SS (left) and 6HB-5*/17/5*-DS (right). (**B**) Schematic of the cylindrical shell method used for calculating the charge density. The density was calculated as $-z\langle n(r)\rangle/(2\pi r\Delta r\Delta l)$, where z is the charge valence of each bead ($-3.2e$ and $-11.8e$ for single- and double-stranded brushes), $\langle n(r)\rangle$ is the number of brush beads within a cylindrical shell of radius r, thickness $\Delta r = 2.4$ nm, and length $\Delta l = 14$ nm centered in the grafted region to avoid ends effects, and $2\pi r\Delta r\Delta l$ is the volume of the cylindrical shell.

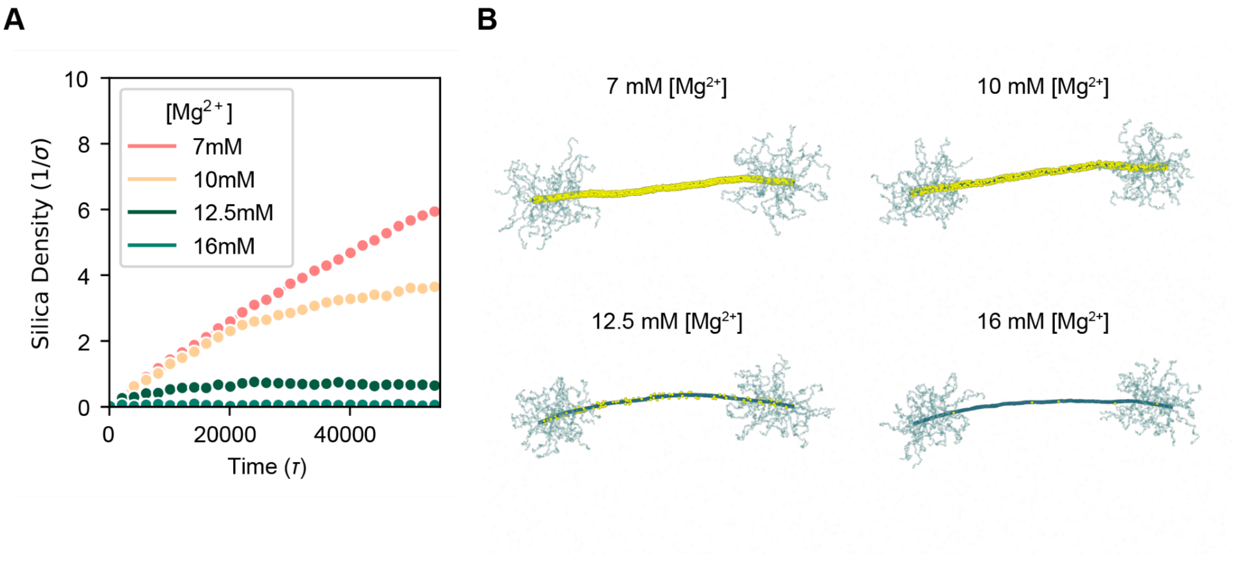

Figure S23. Effect of magnesium concentration on silicification. (**A**) Number density of silica precursors accumulated on the surface of 6HB-5*/17/5*-SS at varying [Mg^{2+}], and (**B**) simulation snapshots of origami showing decreased condensation of silica with increasing [Mg^{2+}]. The size of the silica precursors is doubled for enhanced visibility. To model the experimental [Mg^{2+}] = 7, 10, 12.5, and 16 mM, we used Debye lengths of κ^{-1} = 2.10, 1.75, 1.57, and 1.39 nm in the Debye-Hückel potential used for treating electrostatic interactions between all charged entities in the system. As [Mg^{2+}] increases, the Debye screening length decreases, causing the electrostatic attraction between oppositely charged entities to become shallower and shorter ranged, leading to lesser condensation of the positively charged silica precursors at the negatively charged 6HB.

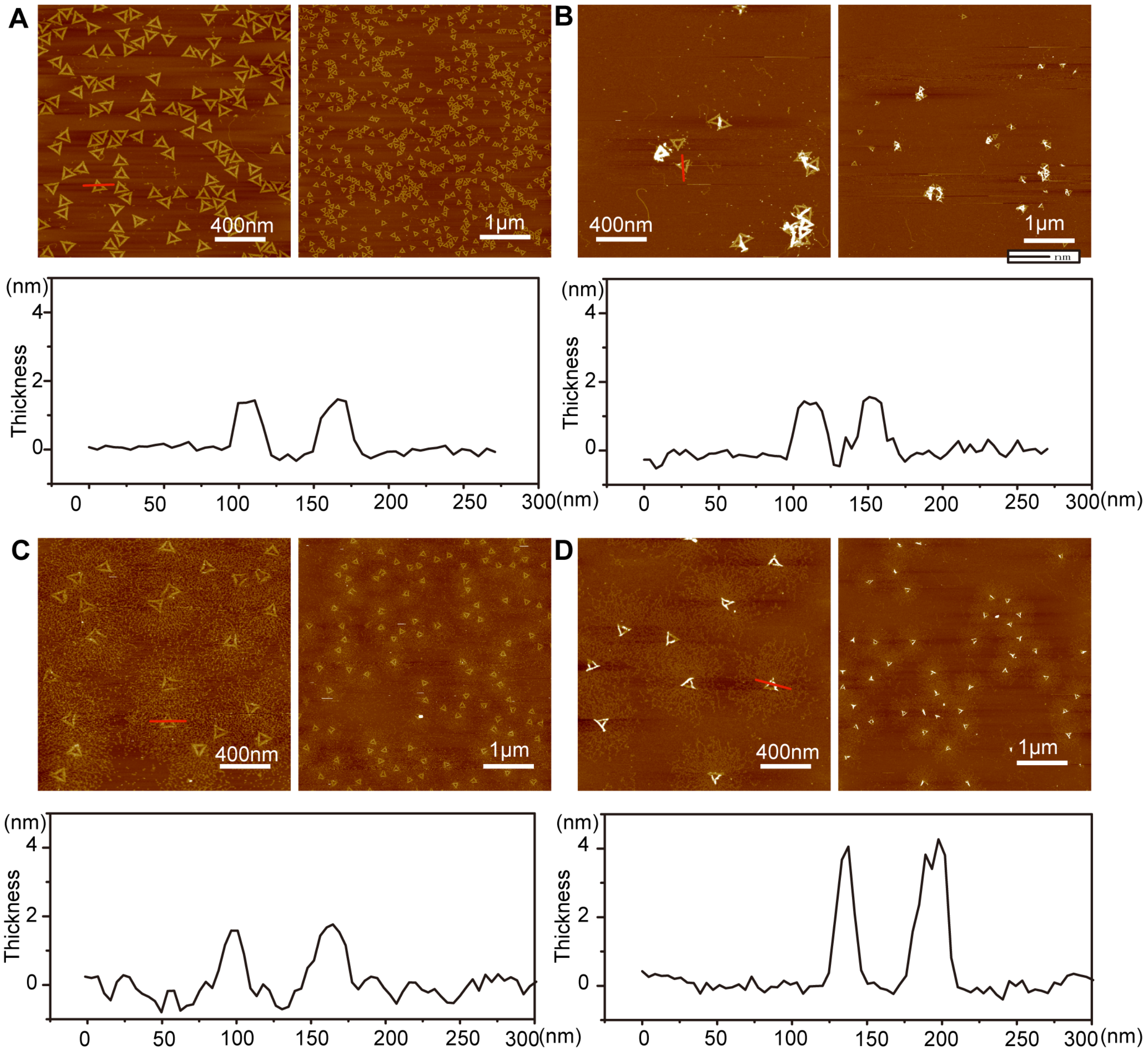

Figure S24. Effect of poly-T on flat DNA structure silicification. AFM images and measured thicknesses of flat triangle without (**A**) and with (**C**) poly-T brushes. AFM images and measured thicknesses of flat triangle without (**B**) and with (**D**) poly-T brushes after silicification. $C_{DNA\ origami}$ = 2 nM, C_{TMAPS} = 2 mM, C_{TEOS} = 1.0 mM, growth time = 4 days.

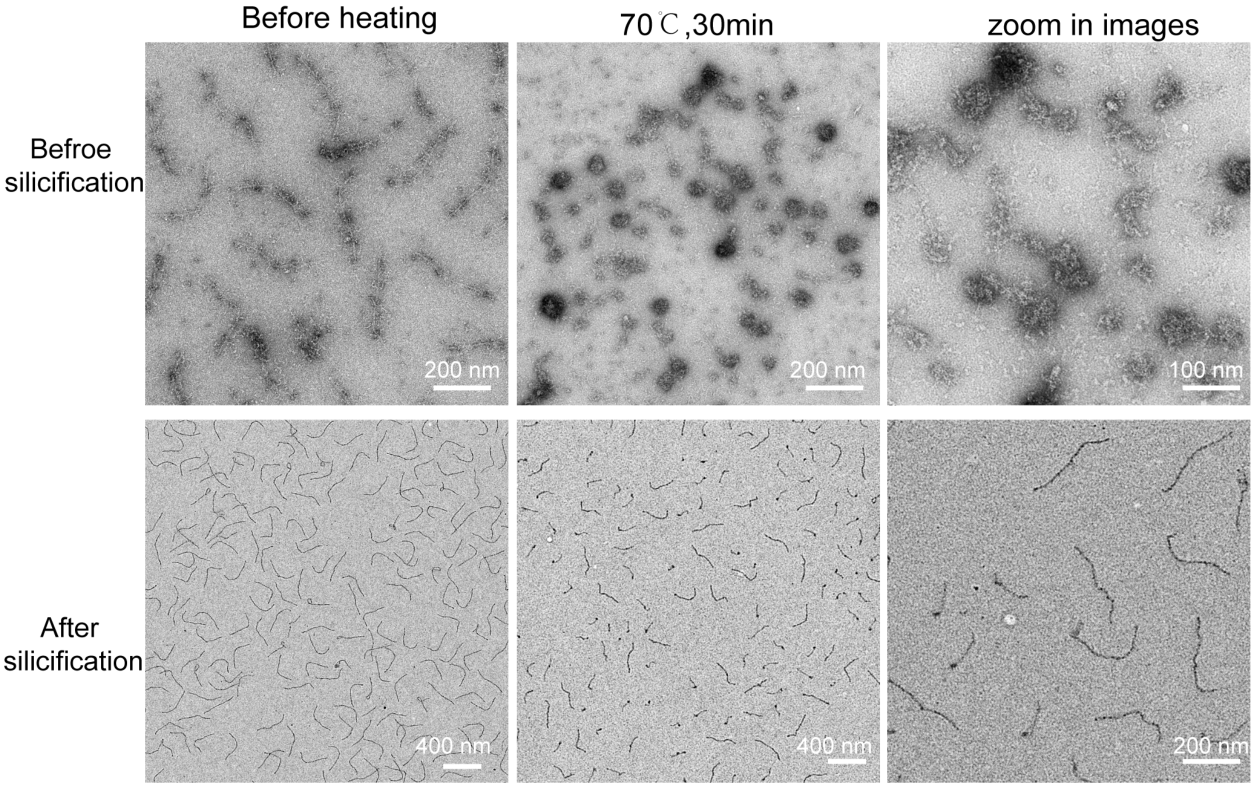

Figure S25. Thermal stability of silicified origami. TEM images of 6HB-27*-SS without and with silicification before and after heating at 70℃ for 30 min.

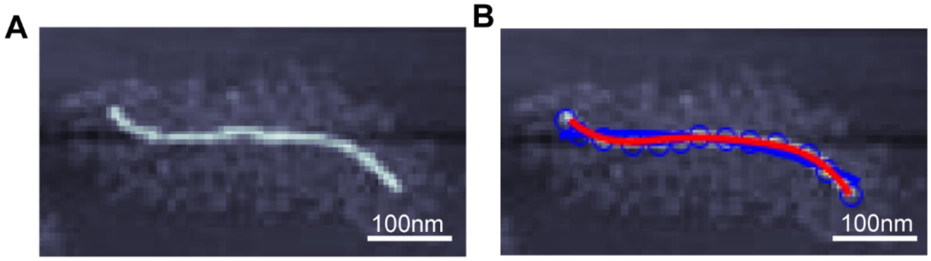

6HB-27*-SS fitted to parametric splines by Easyworm 1

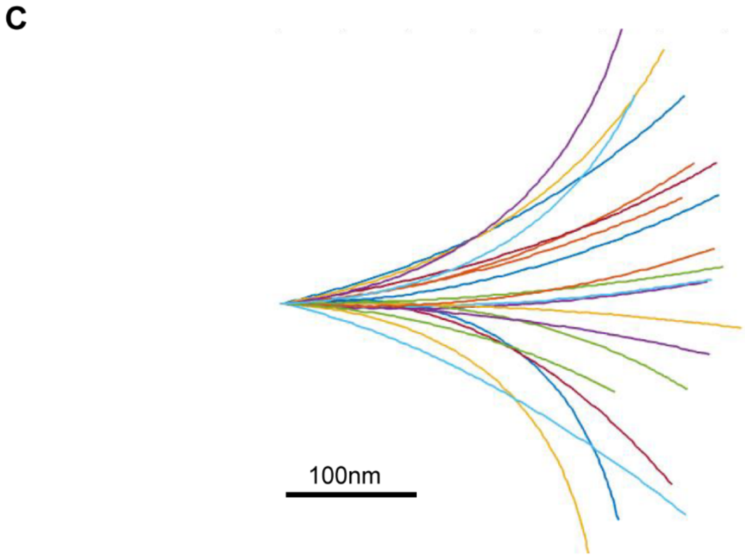

Fluctuations in shape processed by Easyworm 2

Figure S26. Easyworm workflow. (**A**) AFM image of 6HB-27*-SS. (**B**) Fitting the contour of 6HB-27*-SS with a parametric spline (red line). (**C**) 6HB-27*-SS plotted with their initial tangents aligned to facilitate visualization. The detailed analysis protocol is provided in the published manuscript (http://www.scfbm.org/content/9/1/16).

Lists of DNA strands

Supplementary Table 1. All staple strands of 6HB.

Domain	Name	Sequences (5'-3')
**1**	**1-1**	ACAACAACCATCGCAGGTCGACTCTAGAGCGATTAAGTTGGG
	**1-2**	CGAAAGACAGCATCGCTCCTTTTGATAAAACTAAAGTACGGT
	**1-3**	GCGAACCAGACCGGGACTTTTTCATGAGTTGCTTTCGAGGTG
	**1-4**	GTCTGGAAGTTTCAGGATGTGCTGCAAGCCTTTGATAGCGAG
	**1-5**	TAACGCCAGGGTTTTGTTTTAAATATGCGAGGTCATTTTTGC
	**1-6**	GCTTGCATGCCTGCCCACGCATAACCGAGTCACCCTCAGCAG
**2**	**2-1**	AATTTCTTAAACAGGACCGTATACGCATTTCGCTATTACGCC
	**2-2**	CTTTGAGGACTAAAAAGCAAACTCCAACTGATTCCCAATTCT
	**2-3**	CCCGAAAGACTTCATAATGCCACTACGAAAAGGCTCCAAAAG
	**2-4**	GCGAACGAGTAGATTCGGTGCGGGCCTCGGCTAGTACCCGTA
	**2-5**	AGCTGGCGAAAGGGTTCCATATAACAGTAGGTCAGGATTAGA
	**2-6**	GCAAGTCCGCTAGCCTTGATACCGATAGAACGGCTACAGAGG
**3**	**3-1**	GAGCCTTTAATTGTTACCGAGCTCGAATCAGGCTGCGCAACT
	**3-2**	ACGGGTAAAATACGAATATCGCGTTTTAAGATACATTTCGCA
	**3-3**	GTCAGAAGCAAAGCAAGAATACACTAAAAATTGCGAATAATA
	**3-4**	AATGGTCAATAACCCGCCATTCGCCATTTCGTAATCATGGTC
	**3-5**	GTTGGGAAGGGCGATTAGTTTGACCATTATTCGAGCTTCAAA
	**3-6**	TAAGGATCCCCGGGATCGGTTTATCAGCGAAGTTTCCATTAA
**4**	**4-1**	ATTTTTTCACGTTGTGTGAAATTGTTATCGCTTCTGGTGCCG
	**4-2**	AACGAAAGAGGCAAGGATTGCATCAAAATTCATTTGGGGCGC
	**4-3**	TGACCATAAATCAAACCAAGCGCGAAACAACAGTTTCAGCGG
	**4-4**	GAGCTGAAAAGGTGCAGCTTTCCGGCACCCGCTCACAATTCC
	**4-5**	GAAACCAGGCAAAGTGTTTAGCTATATTAGATTAAGAGGAAG
	**4-6**	ATAGCTGTTTCCTGAAAATCTCCAAAAAAGGCACCAACCTAA
**5**	**5-1**	AGTGAGAATAGAAACCGGAAGCATAAAGATCGGCCTCAGGAA
	**5-2**	CCCCCAGCGATTATAAATCAGGTCTTTAAATAGTAGTAGCAT
	**5-3**	TGAATCCCCCTCAACTGATAAATTGTGTAGTAAATGAATTTT
	**5-4**	TAACATCCAATAAAGGGACGACGACAGTTGTAAAGCCTGGGG
	**5-5**	GATCGCACTCCAGCGCATCAATTCTACTCCCTGACTATTATA
	**5-6**	ACACAACATACGAGGGAACAACTAAAGGACACTCATCTTTGA
**6**	**6-1**	CTGTATGGGATTTTGCTAACTCACATTAATCGTAACCGTGCA
	**6-2**	ATTTGTATCATCGCATGCTTTAAACAGTCAAAGAATTAGCAA
	**6-3**	CTGGATAGCGTCCATAGCCGGAACGAGGGTTAGCGTAACGAT
	**6-4**	AATTAAGCAATAAAGTGTAGATGGGCGCATTGCGTTGCGCTC
	**6-5**	TCTGCCAGTTTGAGTCATACAGGCAAGGTCAGAAAACGAGAA
	**6-6**	TGCCTAATGAGTGAGCTAAACAACTTTCAAAGTACAACGGAG

**7**	**7-1**	CTAAAGTTTTGTCGAGTCGGGAAACCTGATTGACCGTAATGG
	**7-2**	TGCTCCATGTTACTATACTGCGGAATCGAGCTAAATCGGTTG
	**7-3**	AAAAGAAGTTTTGCACTGACCAACTTTGACAAACTACAACGC
	**7-4**	TACCAAAAACATTAGGAACAAACGGCGGTCGTGCCAGCTGCA
	**7-5**	GATAGGTCACGTTGGCCTCAGAGCATAATCATAAATATTCAT
	**7-6**	ACTGCCCGCTTTCCTCTTTCCAGACGTTCGAAATCCGCGACC
**8**	**8-1**	CTGTAGCATTCCACAACGCGCGGGGAGACGAGTAACAACCCG
	**8-2**	CATAAGGGAACCGACAGAGGGGGTAATATTTTGCGGGAGAAG
	**8-3**	TACCAGACGACGATCAGGCGCATAGGCTAATAGGAACCCATG
	**8-4**	CCTTTATTTCAACGACATTAAATGTGAGGGCGGTTTGCGTAT
	**8-5**	TCGGATTCTCCGTGTGACCCTGTAATACGTAAAATGTTTAGA
	**8-6**	TTAATGAATCGGCCAGACAGCCCTCATACGCAGACGGTCAAT
**9**	**9-1**	TACCGTAACACTGAGTTTTTCTTTTCACCTGGCCTTCCTGTA
	**9-2**	AACGGTGTACAGACAAAAACCAAAATAGTTTAGAACCCTCAT
	**9-3**	ATTACGAGGCATAGCAAGAACCGGATATACCCTCAGAGCCAC
	**9-4**	ATATTTTAAATGCAAAAATAATTCGCGTCAGTGAGACGGGCA
	**9-5**	GCCAGCTTTCATCACAAGGATAAAAATTCGAGAGGCTTTTGC
	**9-6**	TGGGCGCCAGGGTGGTTTCGTCACCAGTAAAGAGGACAGATG
**10**	**10-1**	CACCCTCATTTTCATTCACCGCCTGGCCCATTTTTTAACCAA
	**10-2**	AAGAGTAATCTTGATAAGAGCAACACTATGTAGGTAAAGATT
	**10-3**	AATACCACATTCAAGCTCATTCAGTGAATTTAGTACCGCCAC
	**10-4**	CAAAAGGGTGAGAATTGTTAAATCAGCTCTGAGAGAGTTGCA
	**10-5**	TAGGAACGCCATCAATGCCTGAGTAATGTCATAACCCTCGTT
	**10-6**	ACAGCTGATTGCCCGGGATAGCAAGCCCGGCTGACCTTCATC
**11**	**11-1**	CCTCAGAACCGCCACTGGTTTGCCCCAGATATTTTGTTAAAA
	**11-2**	AACGTAACAAAGCTCTAATGCAGATACACAAATCACCATCAA
	**11-3**	AACATTATTACAGGAACGAGTAGTAAATATAAGTATAGCCCG
	**11-4**	TATGATATTCAACCAATTGTAAACGTTACAGGCGAAAATCCT
	**11-5**	TTCGCATTAAATTTAGGCCGGAGACAGTTAACGCCAAAAGGA
	**11-6**	GCAAGCGGTCCACGCCCTCAGAACCGCCTCATTACCCAAATC
**12**	**12-1**	GAATAGGTGTATCACCGAAATCGGCAAAACAGGAAGATTGTA
	**12-2**	ACGAGAAACACCAGTAGAAAGATTCATCATTAATGCCGGAGA
	**12-3**	TTGGGAAGAAAAATTAATCATTGTGAATTTGCTCAGTACCAG
	**12-4**	GGGTAGCTATTTTTGAAAAGCCCCAAAAATCCCTTATAAATC
	**12-5**	TAAGCAAATATTTAGTTCTAGCTGATAAAGTTGAGATTTAGG
	**12-6**	GTTTGATGGTGGTTCCGTACTCAGGAGGTAAGGCTTGCCCTG
**13**	**13-1**	GCGGATAAGTGCCGAGATAGGGTTGAGTCAATCATATGTACC
	**13-2**	TTTAATTTCAACTTCTACGTTAATAAAAGGCTATCAGGTCAT
	**13-3**	CAATCCAAAAATCGAGAAACAATAACGGGACTCCAACGTCAA
	**13-4**	TGCCTGAGAGTCTGTAAAACTAGCATGTGTTGTTCCAGTTTG

	**13-5**	CCGGTTGATAATCAGAGAGATCTACAAACGAACTAACGGAAC
	**13-6**	AAAAGAATAGCCCGTCGAGAGGGTTGATTGGGCTTGAGATGG
**14**	**14-1**	CAAGAGAAGGATTATATTAAAGAACGTGATTCGCCTGATTCGATGAACG
	**14-2**	TTAAGAACTTTTTTTGTTTAACGTCAAAAGGCTGAGACTCCT
	**14-3**	CTAATTTGCCAGTTATAACATAAAAACACCTATTTCGGAACC
	**14-4**	AAGTTACAATAAGAAACGAGGCTCATTATACCAGTCAGGACG
	**14-5**	GTAATCGGAGCAAACAAGAGAATGCTTTGAATACC
	**14-6**	GAACAAGAGTCCACGGATTAGCGGGGTTTACCTTATGCGATT
**15**	**15-1**	TATTATTCTGAAACTCTATCATGGTTGCATACAGTAACAGTA
	**15-2**	CCTTTACAGAGAGAACAAAATAAACAGCATTCATTTCAATTA
	**15-3**	TATCCTGAATCTTATGAACACCCTGAACGCCTTGAGTAACAG
	**15-4**	CCTGAGCAAAAGAAAACGTCAGATGAATTTTGACGAGCACGT
	**15-5**	CCTTTTACATCGGGCGCAGAGGCGAATTCATATTATTTATCC
	**15-6**	AGGGCGAAAAACCGATGAAAGTATTAAGAATGAAAATAGCAG
**16**	**16-1**	TGCCCGTATAAACATCGTTAGAATCAGATAAAGAAATTGCGT
	**16-2**	ACGGGAGAATTAACCCAACGCTAACGAGCATCAAGAAAACAA
	**16-3**	TTAAATCAAGATTATCAGAGAGATAACCTGATACAGGAGTGT
	**16-4**	AATTAATTACATTTACGTAAAACAGAAAGCGGAGCTAAACA
	**16-5**	AGATTTTCAGGTTTGATGATGAAACAAACGTCTTTCCAGAGC
	**16-6**	ATAACGTGCTTTCCGTTAATGCCCCCTGGGGAAGCGCATTAG
**17**	**17-1**	ACTGGTAATAAGTTGGGATTTTAGACAGTAGAACCTACCATA
	**17-2**	ATTGAGCGCTAATAGTTGCTATTTTGCAAATTACCTTTTTTA
	**17-3**	GCGTTTTAGCGAACAGAGCAAGAAACAATCTGAATTTACCGT
	**17-4**	ATGGAAACAGTACAAATAATGGAAGGGTGAACGGTACGCCAG
	**17-5**	TCAAAATTATTTGCAACAATTTCATTTGCCCAGCTACAATTT
	**17-6**	GGAGGCCGATTAAATTAACGGGGTCAGTAAAGTCAGAGGGTA
**18**	**18-1**	TCCAGTAAGCGTCATTTTTATAATCAGTAATCCTGATTGTTT
	**18-2**	TAAGCCCAATAATACTCCCGACTTGCGGTGAGTGAATAACCT
	**18-3**	AATCAGATATAGAACCCTTTTTAAGAAAAACAAATAAATCCT
	**18-4**	TGCTTCTGTAAATCCAATTCATCAATATGAGGCCACCGAGTA
	**18-5**	GGATTATACTTCTGTAAATCAATATATGGAGGTTTTGAAGCC
	**18-6**	AATCCTGAGAAGTGTACATGGCTTTTGACACAAGAATTGAGT
**19**	**19-1**	CATTAAAGCCAGAATCACGCAAATTAACCATCATATTCCTGA
	**19-2**	CTATCTTACCGAAGGGCTTATCCGGTATATTTTCCCTTAGAA
	**19-3**	TATTTTCATCGTAGAGAAGGAAACCGAGAGGAGGTTGAGGCA
	**19-4**	TCCTTGAAAACATAAGGAGCGGAATTATCGTTGTAGCAATAC
	**19-5**	TTATCAGATGATGGGTCGCTATTAATTATCTAAGAACGCGAG
	**19-6**	AAAGAGTCTGTCCATGGAAAGCGCAGTCTGAAATAGCAATAG
**20**	**20-1**	GGTCAGACGATTGGATAACATCACTTGCCATTTTGCGGAACA
	**20-2**	CGAACAAAGTTACCGAATCATTACCGCGTAAGACGCTGAGAA

	**20-3**	TAAACCAAGTACCGAGAACTGGCATGATGCCGCCACCAGAAC
	**20-4**	GAGTCAATAGTGAATTGAGTAACATTATCTGAGTAGAAGAAC
	**20-5**	AAGAAACCACCAGAGCGATAGCTTAGATCCCAATAGCAAGCA
	**20-6**	TTCTTTGATTAGTACCTTGATATTCACAAGTAAGCAGATAGC
**21**	**21-1**	CACCACCAGAGCCGTTGCTGGTAATATCTTGCCCGAACGTTA
	**21-2**	ACGGAATACCCAAACACTCATCGAGAACAGGTCTGAGAGACT
	**21-3**	CAATAATCGGCTGTCAAACGTAGAAAATGCCACCCTCAGAAC
	**21-4**	ACCTTTTTAACCTCTCGTATTAAATCCTCAGAACAATATTAC
	**21-5**	TTAATTTTAAAAGTTTTATCAAAATCATAAGCAAGCCGTTTT
	**21-6**	TCAAACTATCGGCCCCGCCAGCATTGACGAAACGCAATAATA
**22**	**22-1**	CGCCACCCTCAGAGACAGGAAAAACGCTTATTAGACTTTACA
	**22-2**	ACGCAGTATGTTAGCTTTCCTTATCATTTTATATAACTATAT
	**22-3**	AAATAATATCCCATGAAACGCAAAGACAACCGGAACCAGAGC
	**22-4**	GTAAATGCTGATGCTGAGGATTTAGAAGCATGGAAATACCTA
	**22-5**	AACAATTCGACAACCGGCTTAGGTTGGGCCAAGAACGGGTAT
	**22-6**	CGCCAGCCATTGCACCACCACCCTCAGATAAGACTCCTTATT
**23**	**23-1**	CACCACCGGAACCGATCGTCTGAAATGGGATTAGAGCCGTCA
	**23-2**	GGCAACATATAAAACCTAATTTACGAGCGACAAAGAACGCGA
	**23-3**	AACGCGCCTGTTTACAATAGAAAATTCAAGCCCCCTTATTAG
	**23-4**	GAAAACTTTTTCAAACTAACAACTAATAATTATTTACATTGG
	**23-5**	ATAGATAATACATTAAATCCAATCGCAAATGTAGAAACCAAT
	**23-6**	CATTTTGACGCTCACCTCCCTCAGAGCCACATACATAAAGGT
**24**	**24-1**	CGTTTGCCATCTTTACACGACCAGTAATGAAGGTTATCTAAA
	**24-2**	TATTTTGTCACAATTCAACAATAGATAAATTTCATCTTCTGA
	**24-3**	TGTCCAGACGACGAGGCGACATTCAACCAGCGTCAGACTGTA
	**24-4**	CCTAAATTTAATGGTGAAAGGAATTGAGAAAAGGGACATTCT
	**24-5**	ATATCTTTAGGAGCATATATTTTAGTTAGTCCTGAACAAGAA
	**24-6**	CAGATTCACCAGTCTCATAATCAAAATCCCACGGAATAAGTT
**25**	**25-1**	GCGCGTTTTCATCGGAACCCTTCTGACCATATCTGGTCAGTT
	**25-1**	GCCAAAGACAAAAGCAATAAACAACATGCGTGTGATAAATAA
	**25-3**	TAATAAGAGAATATGGAAATTATTCATTAGCAGCACCGTAAT
	**25-4**	GGCGTTAAATAAGACAAACCCTCAATCATGAAAGCGTAAGAA
	**25-5**	GGCAAATCAACAGTTTTGAAATACCGACTTCAGCTAATGCAG
	**25-6**	GGCCAACAGAGATAGCATTTTCGGTCATTATGGTTTACCAGC
**26**	**26-1**	CAGTAGCGACAGAAAATATTTTTGAATGAAGCATCACCTTGC
	**26-2**	AGGTAAATATTGACAAAGTACCGACAAACATAATTACTAGAA
	**26-3**	CAACGCCAACATGTGAGCCATTTGGGAAACCATTAGCAAGGC
	**26-4**	AAAGCCTGTTTAGTAAATGAAAAATCTAGCTATTAGTCTTTA
	**26-5**	TGAACCTCAAATATATAAACACCGGAATAGGTAAAGTAATTC
	**26-6**	TACGTGGCACAGACTCAAGTTTGCCTTTGATTGAGGGAGGGA

**27**	**27-1**	CGGAAACGTCACCAAGCCCTAAAACATCCAACAGTGCCACGC
	**27-2**	ACCGTCACCGACTTAATTTAGGCAGAGGACAAATTCTTACCA
	**27-3**	ACCGTCACCGACTTAATTTAGGCAGAGGACAAATTCTTACCA
	**27-4**	GTATAAAGCCAACGATTAACACCGCCTGGCCATTAAAAATAC
	**27-5**	TGAGAGCCAGCAGCATCATATGCGTTATCATTTTCGAGCCAG
	**27-6**	ATGCGCGAACTGATATGAAACCATCGATAAAGGTGAATTATC

Note: Sequences of other structures with initiators are based on the sequences of 6HB, only replacing the specific staples at prescribed position. For instance, 6HB-18*/9-SS structure, we only extend eight thymine at 3' end of domain 1-18; we stretch out initiators at 3'ends of domain 1-9, named 6HB-9*/18-SS structure. 6HB-5*/17/5*-SS structure, we add eight thymine at the 3'end of domain 1-5, and 23-27; we add initiators at 3 ends of all the staples to have full poly T growth (6HB-27*-SS).

Reference

1. Ober, M. F.; Baptist, A.; Wassermann, L.; Heuer-Jungemann, A.; Nickel, B., In situ small-angle X-ray scattering reveals strong condensation of DNA origami during silicification. *Nature communications* **2022,** *13* (1), 5668.
2. Tang, L.; Navarro, L. A., Jr.; Chilkoti, A.; Zauscher, S., High-Molecular-Weight Polynucleotides by Transferase-Catalyzed Living Chain-Growth Polycondensation. *Angew. Chem. Int. Ed. Engl.* **2017,** *56* (24), 6778-6782.
3. Arya, G.; Zhang, Q.; Schlick, T., Flexible histone tails in a new mesoscopic oligonucleosome model. *Biophys. J.* **2006,** *91* (1), 133-150.
4. DeLuca, M.; Sensale, S.; Lin, P.-A.; Arya, G., Prediction and Control in DNA Nanotechnology. *ACS Applied Bio Materials* **2023**.
5. Liu, X. G.; Zhang, F.; Jing, X. X.; Pan, M. C.; Liu, P.; Li, W.; Zhu, B. W.; Li, J.; Chen, H.; Wang, L. H.; Lin, J. P.; Liu, Y.; Zhao, D. Y.; Yan, H.; Fan, C. H., Complex silica composite nanomaterials templated with DNA origami. *Nature* **2018,** *559* (7715), 593-598.
6. Carcouët, C. C. M. C.; van de Put, M. W. P.; Mezari, B.; Magusin, P. C. M. M.; Laven, J.; Bomans, P. H. H.; Friedrich, H.; Esteves, A. C. C.; Sommerdijk, N. A. J. M.; van Benthem, R. A. T. M.; de With, G., Nucleation and Growth of Monodisperse Silica Nanoparticles. *Nano Letters* **2014,** *14* (3), 1433-1438.
7. Kremer, K.; Grest, G. S., Dynamics of entangled linear polymer melts: A molecular-dynamics simulation. *The Journal of Chemical Physics* **1990,** *92* (8), 5057-5086.
8. DeLuca, M.; Ye, T.; Poirier, M.; Ke, Y.; Castro, C.; Arya, G., Mechanism of DNA origami folding elucidated by mesoscopic simulations. *bioRxiv* **2023**, 2023.06.20.545758.
9. Ambia-Garrido, J.; Vainrub, A.; Pettitt, B. M., A model for structure and thermodynamics of ssDNA and dsDNA near a surface: A coarse grained approach. *Computer physics communications* **2010,** *181* (12), 2001-2007.
10. Thompson, A. P.; Aktulga, H. M.; Berger, R.; Bolintineanu, D. S.; Brown, W. M.; Crozier, P. S.; in't Veld, P. J.; Kohlmeyer, A.; Moore, S. G.; Nguyen, T. D., LAMMPS-a flexible simulation tool for particle-based materials modeling at the atomic, meso, and continuum scales. *Comput. Phys. Commun.* **2022,** *271*, 108171.

11. Stukowski, A., Visualization and analysis of atomistic simulation data with OVITO–the Open Visualization Tool. *Modell. Simul. Mater. Sci. Eng.* **2009,** *18* (1), 015012.
